# Identification of the Key Differential Transcriptional Responses of Human Whole Blood Following TLR2 or TLR4 Ligation *In-Vitro*

**DOI:** 10.1371/journal.pone.0097702

**Published:** 2014-05-19

**Authors:** Simon Blankley, Christine M. Graham, Ashleigh Howes, Chloe I. Bloom, Matthew P. R. Berry, Damien Chaussabel, Virginia Pascual, Jacques Banchereau, Marc Lipman, Anne O’Garra

**Affiliations:** 1 Division of Immunoregulation, MRC National Institute for Medical Research, London, United Kingdom; 2 Department of Respiratory Medicine, Imperial College Healthcare NHS trust, London, United Kingdom; 3 Baylor Institute for Immunology Research/ANRS Center for Human Vaccines, INSERM, Dallas, Texas, United States of America; 4 Systems Immunology, Benaroya Research Institute, Seattle, Washington, United States of America; 5 The Jackson Laboratory for Genomic Medicine, Farmington, Connecticut, United States of America; 6 Department of Respiratory Medicine, Royal Free London NHS Foundation Trust, University College London, London, United Kingdom; 7 Department of Medicine, National Heart and Lung Institute, Imperial College, London, United Kingdom; DRFZ, Germany

## Abstract

The use of human whole blood for transcriptomic analysis has potential advantages over the use of isolated immune cells for studying the transcriptional response to pathogens and their products. Whole blood stimulation can be carried out in a laboratory without the expertise or equipment to isolate immune cells from blood, with the added advantage of being able to undertake experiments using very small volumes of blood. Toll like receptors (TLRs) are a family of pattern recognition receptors which recognise highly conserved microbial products. Using the TLR2 ligand (Pam3CSK4) and the TLR4 ligand (LPS), human whole blood was stimulated for 0, 1, 3, 6, 12 or 24 hours at which times mRNA was isolated and a comparative microarray was undertaken. A common NFκB transcriptional programme was identified following both TLR2 and TLR4 ligation which peaked at between 3 to 6 hours including upregulation of many of the NFκB family members. In contrast an interferon transcriptional response was observed following TLR4 but not TLR2 ligation as early as 1 hour post stimulation and peaking at 6 hours. These results recapitulate the findings observed in previously published studies using isolated murine and human myeloid cells indicating that *in vitro* stimulated human whole blood can be used to interrogate the early transcriptional kinetic response of innate cells to TLR ligands. Our study demonstrates that a transcriptomic analysis of mRNA isolated from human whole blood can delineate both the temporal response and the key transcriptional differences following TLR2 and TLR4 ligation.

## Introduction

Microarray analysis is increasingly being used to advance our understanding of the complex transcriptional responses generated downstream of an experimental perturbation or during a disease state [1], [2]. Using modern microarray platforms it is possible to measure the expression of over 40,000 mRNA transcripts, encompassing all of the known functional human genome. By sequentially sampling over time, the temporal dynamics of the transcriptional response to a given event can be delineated and depending on the scale of the response many hundreds or thousands of significantly differentially expressed genes may be identified. To better understand and interpret this complex data bioinformatics tools have been developed that take advantage of known biological relationships which may influence gene expression. Using these tools it is possible to use an unbiased methodology to determine distinct classes of differentially regulated genes as well as potentially important transcriptional regulatory pathways or networks which may change when exposed to a given stimulus [2]–[4].

Toll-like receptors (TLR) are a family of pattern recognition receptors (PRR) which recognise highly conserved microbial products. In humans 10 functional TLRs have been described including TLR2 and TLR4 which are expressed on the cell surface and recognise conserved bacterial products [5]. TLR2 recognises lipoteichoic acids of Gram-positive bacteria and bacterial lipo-proteins whereas TLR4 recognises lipopolysaccharides (LPS) of Gram-negative bacteria [5], [6]. TLR2, TLR3 and TLR4 are expressed and functional in a wide variety of cells found in human whole blood including dendritic cells (DC) and monocytes [6]. Early in the immune response to a pathogen, ligation of TLRs induces gene transcription leading to inflammation, tissue repair and the initiation of adaptive immunity [6], [7]. All TLRs utilise the MyD88 adaptor molecule except for TLR3 which uses the TRIF-TRAM adaptor molecules only. TLR2 and TLR4 both use the MyD88-TIRAP adaptor molecules. Additionally TLR4 also uses the TRIF-TRAM adaptor molecules [5]–[7]. Signalling via the adaptor molecule MyD88 results in activation of transcription factors such as NFκB and AP-1 [5]–[7], whereas signalling via the TRIF-TRAM adaptors leads to activation of the interferon regulatory transcription factors (IRF) including IRF3 [6], [8]. IRF3 is constitutively expressed and its activation by ligation of TLR4 or TLR3 results in to induction of IFNβ (amongst other cytokines), which via the IFNαβ receptor leads to positive feedback regulation of type 1 interferon (IFN) genes including the type 1 IFN inducible transcription factor IRF7 [8]. There is also a differential temporal transcriptional response following NFκB activation in macrophages, which can be broadly categorised into three phases: early primary response, late primary response and secondary response genes. This differential activation of sets of genes depends on their chromatin status and the potential need for remodelling of the target genes which can lead to the differential kinetics of induction [7], [9]. Much of the knowledge we have gained of these signalling mechanisms are derived from studying TLR ligation of monocytes, macrophages or DCs from mice or humans.

A study collectively analysing data from multiple published papers from both human and murine macrophage transcriptional studies identified a group of genes upregulated following both TLR2 and TLR4 stimulation, which were predicted to be regulated by NFκB. Additionally a separate IFN-sensitive response element (ISRE) regulated set of genes expression was seen to be upregulated in the TLR4 (and TLR3) stimulations but not the TLR2 stimulation [10]. Importantly the temporal kinetics of these two groups of genes differed, with the genes predicted to be regulated by NFκB peaking in expression earlier than the predicted ISRE regulated genes. In addition, genes thought to be regulated by both ISRE and NFκB had a greater magnitude of induction though similar temporal kinetics to the genes regulated only by ISRE [10]. However this study had limited time points available for the TLR2 stimulations and so the majority of the temporal analysis was undertaken using LPS stimulated samples. Another comparative study including TLR2, TLR3 and TLR4 stimulation of murine primary DCs identified an “inflammatory programme” mediated by TLR2 ligation and an “anti-viral programme” mediated by TLR3 ligation. TLR4, owing to its use of both MyD88-TIRAP and TRIF-TRAM adaptor molecules, resulted in the activation of both “inflammatory” and “anti-viral” programmes [11]. The “inflammatory” programme was enriched for genes predicted to be regulated by NFκB (RELA, NFκB1, NFκBIZ) and the “anti-viral” programme was enriched for genes predicted to be regulated by the transcription factors STAT1, STAT2, STAT4, IRF8 and IRF9 [11]. Study of the temporal transcriptomic response following TLR4 ligation in human and murine macrophages has also been used to specifically analyse the expression of transcription factors [12]–[14]. For example in LPS stimulated human macrophages the majority of transcription factors whose expression was seen to change had done so by 2 hours. Groups of transcription factors peaked in expression at different times, coinciding with the transcriptional peaks of the genes they were predicted to regulate following TLR stimulation in both murine and human macrophages [12], [15]. However studies in isolated cells may not reflect the overall host response to TLR ligation, since interaction will occur between different cell types, leading to a complex interplay of autocrine and paracrine signalling events resulting in differentiation, proliferation, cell trafficking and further chemokine/cytokine production and feedback loops for positive and negative effects on gene regulation.

Transcriptomic studies following *in vivo* TLR4 ligand administration have also previously been undertaken [16]–[18]. From human whole blood leukocytes obtained 0, 2, 4, 6, 9 and 24 hours following *in vivo* administration of LPS, it was shown that mRNA expression of proinflammatory chemokines and cytokines (TNF, IL1A, IL1B, CXCL1, CXCL2, CCL2, CXCL8 and CXCL10) peaked at 2 to 4 hours after LPS administration, whereas the cytokine IL10 was maximal at 6 hours. In this study the transcription factors NFKB1, NFKB2, RELA and RELB were significantly expressed and seen to peak after the cytokines and chemokines. The peak time for transcription factors including the STAT (signal transducer and activator of transcription) and IRF genes was at 4 to 6 hours [16], [19]. Analysis of mRNA isolated from circulating human neutrophils after 0, 2, 4 and 6 hours following LPS administration revealed significant upregulation of the TNF signalling pathway and NFκB genes such as NFκB1 and NFκB2 by 2 hours [18]. Although it is possible to undertake certain human *in vivo* experiments, studies are limited by ethical and practical considerations.

Whole blood comprises cells of both the innate and adaptive immune system. Therefore the use of whole blood for *in vitro* studies has the potential advantage over isolated cell populations as these different components may have a differential response to stimulation. Autocrine and paracrine signalling between the differing cell populations may result in a response of the whole system that potentially better reflects the *in-vivo* response. Additionally whole blood potentially has advantages over PBMC, DCs or monocyte derived macrophages since it can be used in situations where it is not possible to obtain large volumes of blood to derive the isolated cell populations. Previously, *In-vitro* human whole blood has been used as a model to study TLR ligation, predominantly with measurement of specific cytokine protein levels [20]–[23] or specific cytokine mRNA levels as the readout [24], [25]. Human whole blood has also been used as a model to assess the whole genome transcriptional response to TLR4 [26] or TLR2 and TLR4 ligands [27], although these studies only looked at one time point with limited analysis.

The objective of our study was to undertake a detailed comparative analysis of the *in vitro* global temporal transcriptional response to TLR4 and TLR2 ligation in human whole blood. To better understand this gene transcriptional response we used a variety of bioinformatics approaches to delineate both the temporal response and the key transcriptional differences resulting from TLR2 and TLR4 ligation and demonstrate that in a whole blood system that the response to TLR stimulation can resemble that previously identified in isolated immune cells.

## Materials and Methods

### Ethics Statement

This study was approved by the Central London 3 Research Ethics Committee (09/H0716/41). All participants gave written informed consent.

### Human Volunteers

Six healthy volunteers (self-reported questionnaire); three male, three female; aged 25–50 years old; of similar ethnic background were recruited into the study. Sixty ml of whole blood from each volunteer was taken at 9 am into 10 ml Vacutainers with sodium heparin 17 international units/ml (BD Vacutainer).

### Whole Blood Cellular Composition

Measured by Celltac Automated Hematology Analyzer (MEK-6400J/K, Nihon Kohden) at 0 hour, volunteer’s results listed Table S1.

### *In vitro* Whole Blood Stimulation

In 24 well plates (Costar 3524, Corning Incorporated), 1 ml of heparinised whole blood was stimulated either in the presence of a final concentration of 200 ng/ml of Pam3CSK4 (Invivogen), 1 ng/ml of LPS (from Salmonella Minnesota R595, Enzo Life Sciences) added in a volume of 100 µl with RPMI-1640 with GlutaMAX (Life Technologies). Media control samples were cultured with the addition of 100 µl of RPMI-1640 with GlutaMAX. Samples were incubated at 37°C, 5% CO_2_ for 0, 1, 3, 6, 12 or 24 hours at which point the contents of the well were thoroughly mixed with 2 mls Tempus Solution (Applied Biosystems/Ambion) to lyse the cells and stabilise the RNA. Samples stored at −80°C until RNA processing.

### Endotoxin Testing

Reagents (excepting LPS and Pam3CSK4) including Sodium heparin Vacutainers were tested for endotoxin contamination by Limulus assay and were found to be endotoxin free (<0.03 EU/ml). Pam3CSK4 was tested by the manufacturer and confirmed to be endotoxin free (<0.001 EU/µg).

### RNA Processing

RNA was isolated using the PerfectPure RNA Blood kit (5-PRIME) according to manufacturer’s instructions. 2.5 µg of isolated RNA was globin RNA reduced using the GLOBINclear 96-well format kit (applied Biosystems/Ambion) according to the manufacturer’s instructions. Isolated and globin reduced RNA quantity was assessed using either Nanodrop 1000 or Nanodrop 8000 spectrophotometer (NanoDrop Products, Thermo Fisher Scientific), RNA quality was assessed using an Agilent 2100 Bioanalyser (Range 6.5–9.5) (Agilent technologies). 200 ng of globin reduced RNA was amplified to generate biotinylated amplified antisense cRNA using the Illumina CustomPrep RNA amplification kit (Applied Biosystems/Ambion). 750 ng of cRNA was hybridized to Illumina Human HT-12 V4 BeadChip arrays (Illumina) and scanned on Illumina iScan. GenomeStudio (Illumina) was used to perform quality control and generate signal intensity. Two samples were excluded from further analysis at this stage as they failed quality control measures (0 hour media control x1, 6 hour media control x1).

### Microarray Analysis

Raw background subtracted data was processed using Genespring V12.6 (Agilent Technologies) and the following principles were applied to all analyses. After background subtraction low signal values (<10) were then set to a threshold of 10, log2 transformed and per chip normalised using 75^th^ percentile shift algorithm. Per-transcript normalisation was undertaken by normalisation to the median of a defined control group. Transcripts were then filtered out if they were not significantly (*p*<0.01) different in intensity value compared to the background in at least 10% of all the samples.

The resulting transcripts were then subjected to statistical filtering (either One-way ANOVA or 2-way ANOVA) with multiple testing correction (Benjamini-Hochberg *p*<0.01), followed by a further filtering of transcripts by fold change (FC) in which transcripts were filtered if less than 1.8 FC different between variables of interest. Expression heatmaps were generated within Genespring V12.6. Heatmap clustering was undertaken using Differential distance metric and Wards linkage rule, unless otherwise stated.

Media controls from 2 volunteers had evidence of activation of inflammatory genes by 3 hours of culture (Figure S1A). This activation persisted in all of the subsequent time points for these individuals (not shown) appears to be revealed in culture, is independent of the individual, and independent of the length of time in transport conditions (Figure S1B). These samples were excluded from the study.

The data discussed in this publication have been deposited in NCBI's Gene Expression Omnibus and are accessible through GEO Series accession number GSE55375.

### *k*-means Clustering

Within Genespring 12.6 the normalised significant transcript lists were separately clustered by *k*-means clustering into 9 clusters using Euclidian distance metric. Number of clusters was chosen by the number of 3^rd^ order branches in the dendrogram from the LPS expression heatmap. Clusters were compared across stimulations using Pearsons Correlation (within Graphpad Prism V6).

### Ingenuity Pathway Analysis (IPA)

The canonical pathway, gene function annotation and upstream analyses were generated through the use of IPA (Ingenuity Systems, www.ingenuity.com). Significant transcripts identified from GX microarray analysis were uploaded into IPA. For time point analyses these lists were filtered by mean FC (>1.8) compared to media control at the time point.

### Type I IFN Regulated List

A list of human type I regulated genes was obtained from the Interferome V2 database (Accessed June 2013) [28].

### Quantitative PCR

From the globin reduced RNA cDNA was synthesised using High Capacity cDNA Reverse Transcription kit (Applied Biosystems), according to the manufacturer’s instructions followed by RNase H (Promega) treatment for 30 min at 37°C. IFNB1, IL1A, IL6, NFKB1, NFKB2, STAT1, STAT2 and IRF7 gene expression were quantified by real-time PCR (7900HT, Applied Biosystems) using the TaqMan system, and normalised to GAPDH mRNA.

Primer probes used were IFNB1 (Hs01077958_s1); IL1A (Hs00174092_m1); IL6 (Hs00985639_m1); NFKB1 (Hs00765730_m1); NFKB2 (Hs010208901); STAT1 (Hs01013996_m1); STAT2 (Hs01013123_m1); IRF7 (Hs01014809_g1); GAPDH (Hs02758991_g1) (all Applied Biosystems).

## Results

### Media Controls

Analysis of the media controls alone over time revealed 377 significantly expressed transcripts over time. The peak of this difference was at 24 hours with 281 transcripts more than 1.8FC different compared to the 0 hour samples (Figure S2A). These differentially expressed transcripts at 24 hours were enriched for inflammatory and metabolic function pathways (Figure S2B). For this reasons all data for stimulations are filtered against the media controls from the time point of interest and fold changes calculated as relative to the media controls at the same time point.

### Identification of Significantly Differentially Regulated Transcripts following LPS and Pam3CSK4 Stimulation

To gain insight into the differential temporal gene expression in response to TLR4 and TLR2 ligation we performed a comparative microarray analysis of LPS and Pam3CSK4 stimulated human whole blood and accompanying media controls over a time course. From four healthy human volunteers 1 ml of heparinised whole blood was stimulated with either the TLR4 ligand LPS (1 ng/ml), the TLR2 ligand Pam3CSK4 (200 ng/ml), (concentrations shown to result in a plateau in previous studies within our laboratory), or incubated only with media as a control. RNA was isolated at 0, 1, 3, 6, 12 and 24 hours for each stimulus or control.

**Figure 1:**
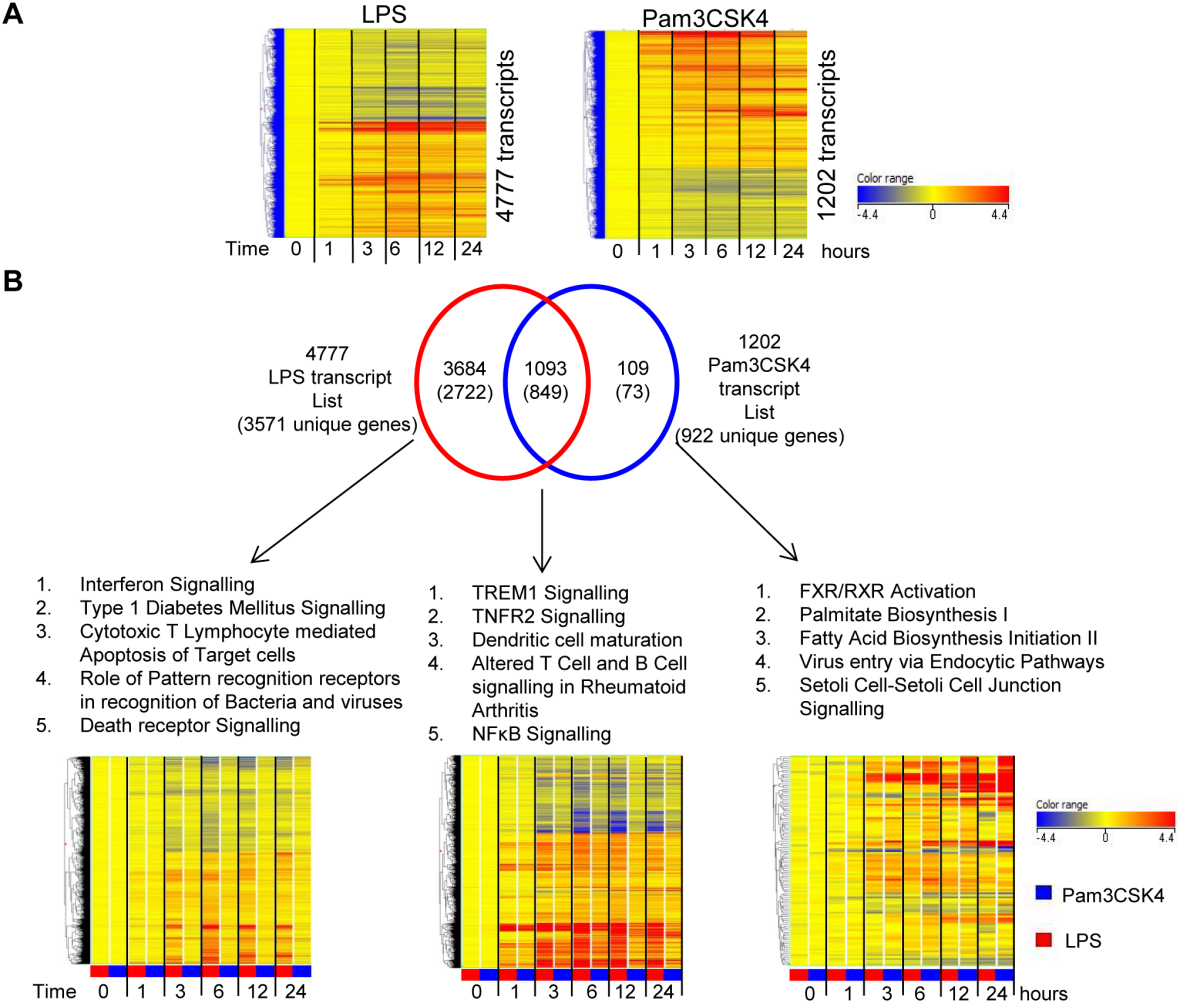
LPS or Pam3CSK4 stimulations results in a differential response in gene expression over time. (**A**) 1 ml of human whole blood from healthy volunteers (N = 4) was stimulated with either Pam3CSK4 (200 ng/ml), LPS (1 ng/ml) or media control for different lengths of time (0, 1, 3, 6, 12 and 24 hours). Stimulations were analysed independently: media control compared to Pam3CSK4 and media control compared to LPS revealed 1202 and 4777 significantly expressed transcripts respectively. Transcripts were identified by normalising expression values to the median of the 0 hour samples, filtering by detection from background, statistical filtering (2 way ANOVA with Benjamini Hochberg multiple testing correction *p*<0.01) and retaining transcripts whose expression was greater than 1.8 FC different between the media control and stimulation samples at one or more time point. (**B**) A Venn diagram of both significant transcript lists. Within the Venn for each subset the number of transcripts is given, with unique genes within IPA in brackets. For transcript lists the top 5 canonical pathways (IPA) are shown as well as a heat map of the normalised expression values of these transcripts for both stimulations over time.

LPS (TLR4) stimulation induced a greater number of differentially regulated transcripts as compared to Pam3CSK4 (TLR2) and had a higher magnitude of response. For this reason the two stimulations were first analysed independently to generate the significant transcript lists compared to media control over the time course after stimulation. LPS stimulation resulted in the differential expression of 4777 transcripts (mapping to 3571 unique genes in IPA) whereas Pam3CSK4 stimulation resulted in only 1202 differentially regulated transcripts (mapping to 922 unique genes in IPA) (Figure 1A). Expression of these transcripts varied over time. Overall 90% of the 1202 significant Pam3CSK4 transcripts were shared with the LPS stimulation (Figure 1B). These shared 1093 transcripts when analysed by canonical pathway analysis (within IPA) were shown to contain TREM, TNF and NFκB signalling amongst the top 5 pathways (ranked by significance). Canonical Pathway analysis of the 3684 transcripts significantly expressed following LPS and not Pam3CSK4 stimulation revealed “IFN signalling” as the most significant pathway.

### *k-*means Clustering Analysis Reveals Similarities and Differences between Stimulations

**Figure 2:**
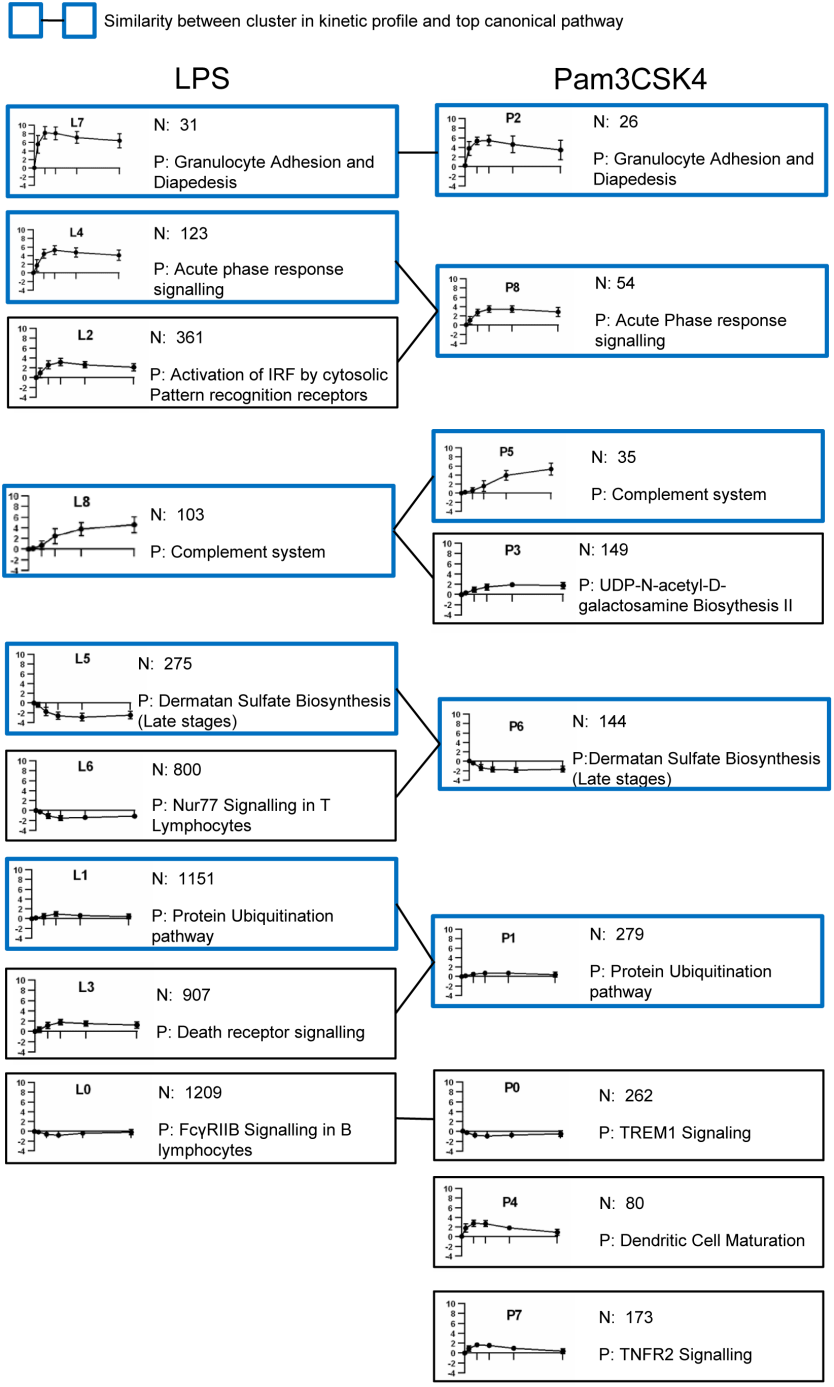
*k*-means clustering of the significant transcript lists reveals similar clusters. Mean normalised expression profile of individual clusters (y axis, ±SD, n = 4) at each time point (x axis; 0, 1, 3, 6, 12 and 24 hours) for each cluster. N: number of transcripts within cluster; P: most significant canonical pathway (IPA). Clusters are grouped by similarity in kinetic profile (Pearsons correlation, Table S2) and top canonical pathway.

*k*-means clustering was applied to cluster the significant transcript lists based on their similarity in expression over time for each stimulation separately (full composition of the clusters listed in EXCEL Files S1 & S2). For each cluster the most significant canonical pathway (IPA) was determined, reflecting gene enrichment within each cluster. The clusters were then compared by their expression profile over time (Pearson correlation, Table S2) and the top canonical pathway to discover similar clusters between LPS (termed L) and Pam3CSK4 (termed P). Based on these criteria ten clusters in response to both stimulations were equivalent. Of the remaining clusters, six shared similar expression profiles but had different top canonical pathways and two clusters (P4 and P7) had no equivalent in the LPS stimulated clusters (Figure 2).

The similar clusters with “Granulocyte Adhesion and Diapedesis” as the top canonical pathway (L7 and P2) were small in terms of numbers of genes which were highly expressed by 1 hour after stimulation with both LPS and Pam3CSK4 and peaked in expression between 3 to 6 hours. 80% of the genes in the P2 cluster were found within the L7 cluster. These common genes were predominantly chemokines and cytokines: C15orf48, CCL2, CCL20, CCL3, CCL3L1, CCL3L3, CCL4L1, CCL4L2, CCL8, CCRL2, CXCL2, EBI3, IL1A, IL6 and TNF.

The “Acute Phase response signalling” clusters (L4 and P8) were characterised by genes involved with inflammatory response. Approximately 60% of the genes in the P8 cluster were also found within the L4 cluster and these common genes included those involved with the inflammatory response: ORM1, ORM2, HAMP, IRAK2, PI3, PTGES, TNFAIP6, TNIP3. In addition, within the L4 cluster but not the P8 cluster were genes involved with IFN regulation: IFI44L, IFIT1, IFIT3, IFNG, OAS3, OASL.

The “Protein Ubiquitination pathway” clusters (L1 and P1) had 104 common genes between stimulations which included heat shock protein genes (HSP90AA1, HSP90AB1, HSPA5, HSPD1, HSPE1 and HSPH1) and Proteasome PA700/20S genes (PSMA1, PSMC3, PSMC4, PSMD1 and PSMD14).

The “Complement system” clusters (in both L8 and P5) were characterised by genes that were upregulated, had their highest expression at 24 hours and included the metallothionein genes (MT1G, MT1H, MT1E, MT1X, MT1M and MT1F) which were amongst the highest expressed genes at 24 hours following both LPS and Pam3CSK4 stimulations (Figure S3). Clusters L5 and P6 contained transcripts that were down regulated over time and were similarly enriched for the pathway “Dermatan Sulfate Biosynthesis (Late stages)”.

Cluster L2, which peaked at 6 hours and was characterised by “Activation of IRF by cytosolic pattern recognition receptors” as the top pathway did not have a similar pathway in the Pam3CSK4 clusters. Examination of the genes within this pathway revealed that there were detected a number of IFN regulated/regulatory genes (DDX58, DHX58, IFIH1, IFIT2, IFNB1, IRF7, STAT2).

Clusters L3, L0, L6 and P4, P7 and P0 were all enriched for immune function pathways although there were no similarities between the stimulations in terms of top significant pathway and kinetic profile. Cluster P3 was enriched for the pathway “UDP-N-acetyl-D-galactosamine Biosythesis II” and there was no similar cluster following LPS stimulation.

### Common and Distinct Changes in Transcription at the Different Time Points

**Figure 3:**
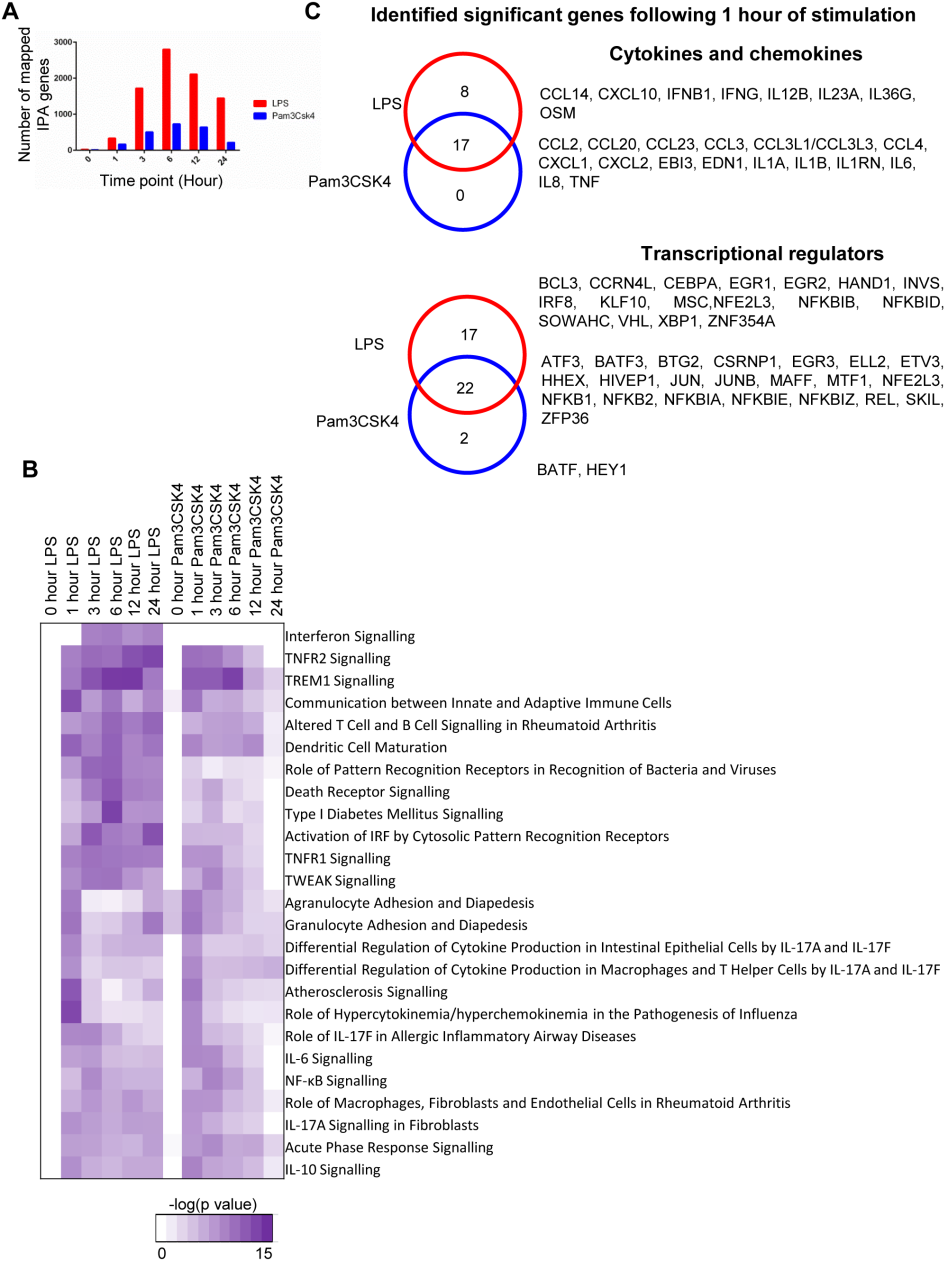
Transcript lists analysed at each time point. (**A**) A graph showing the number of genes from the respective significant transcript lists (4777 LPS and 1202 Pam3CSK4 lists) at each time point which are more than 1.8 FC different compared to the media control at that time point. (**B**) The significantly expressed transcript lists (1202 Pam3CSK4 transcript list and 4777 LPS transcript list) were analysed in IPA. For each time point only genes whose expression were 1.8 FC different from the media control at that time point were taken into consideration. Shown is a heatmap of pathway significance of the top 25 IPA canonical pathways for each time point where significance criteria met (Fishers Exact test *p*<0.01). The IPA canonical pathways were chosen by identifying from the LPS stimulation analyses the top 25 most significant pathways across the time points (mean –log *p* value) and then compared to Pam3CSK4. (**C**) Venn diagrams of cytokine/chemokines and transcriptional regulators identified using IPA gene functional classification from LPS 4777 and Pam3CSK4 1202 transcript lists with mean expression greater than 1.8 FC different to media control at 1 hour. Listed adjacent to the Venn diagrams are the genes from each subset.

To better understand the temporal response we undertook analysis at each time point using the significant transcript lists. Significantly expressed transcripts at each time point were identified by comparing the mean expression of the differentially regulated transcripts in response to LPS and Pam3CSK4 stimulation at each time point to the media control at that time point and filtering those transcripts which were less than 1.8 FC different to the media control (full listings of identified transcripts given as EXCEL Files S3 & S4). From this it was observed that the peak of the transcriptional response compared to media control occurred at 6 hours for both LPS and Pam3CSK4 stimulations (Figure 3A). This differential transcriptional response was still evident at 24 hours following *in vitro* LPS stimulation of whole blood.

At each time point the per-time point transcript lists were analysed by canonical pathway analysis within IPA. The top 25 significant pathways (by mean –log *p* value of the LPS time point pathways and compared to Pam3CSK4) are shown (Figure 3B). This analysis revealed a large number of canonical pathways with similar kinetics of significance in both LPS and Pam3CSK4 stimulations, with the exception of the “IFN Signalling” pathway where there was a clear difference in significance between stimulations.

The pathways that were most significant at 1 hour and then diminished in significance over time were “Agranulocyte Adhesion and Diapedesis”, “Granulocyte Adhesion and Diapedesis”, “Differential Regulation of cytokines production in Epithelial Cells by IL-17A and IL-17F”, “Differential regulation of cytokines production in macrophages and T-Helper Cells by IL-17A” and the pathway “Role of Hypercytokinemia/hyperchemkinemia”. All the genes contributing to these pathways at this time point were chemokine and cytokine genes. “IL-10 signalling”, “Communication between Innate and Adaptive Immune cells” and “Atherosclerosis Signalling” pathways were also most significant at 1 hour and then diminished in significance over time. In addition to cytokine and chemokines genes the “Atherosclerosis Signalling” and “Communication between Innate and Adaptive Immune cells” pathways had CD40, ICAM1, ORM1 and ORM2 as being significantly expressed within the pathways at 1 hour following LPS and Pam3CSK4 stimulation. The “IL-10 signalling” pathway in addition to chemokines and cytokines had the transcription factors, JUN, NFKB1, NFKB2, NFKBIA, NFKBIE as well as the gene SOCS3 within the pathway.

The pathways which were most significant at 3 hours following both LPS and Pam3CSK4 stimulation were “NFκB Signalling” and “IL-6 Signalling”. There were a number of pathways that were significant by 1 hour and remained significant following both LPS and Pam3CSK4 stimulations: “TNFR2 signalling”, “TREM1 signalling”, Altered T cell and B Cell signalling in Rheumatoid arthritis” and “Dendritic cell maturation” pathways.

The “IFN signalling” pathway in response to LPS was only significant (*p*<0.01) from 3 hours onwards and was most significant at 6 hours (Figure 3B). There was a clear difference between the level of significance of the IFN signalling pathway following LPS and Pam3CSK4 stimulation, with the “IFN signalling” pathway not reaching the threshold of significance (*p*<0.01) at any time point following Pam3CSK4 stimulation.

The significantly expressed genes at 1 hour were analysed, as from the canonical pathway analysis we had identified enrichment of cytokines and chemokines genes at this time point. In addition this was the earliest measured time point following stimulation, when autocrine and paracrine signalling leading to induction of mRNA should be at its minimum and, 334 LPS and 165 Pam3CSK4 genes respectively were more than 1.8 FC different to media control at 1 hour. Cytokines/chemokines and transcriptional regulators (identified by IPA gene function annotation) combined accounted for approximately 20% of the significantly expressed genes at this time point. There was a large degree of overlap between stimulations of the significant cytokines/chemokines at 1 hour, with all of the 17 significant cytokines/chemokines genes from the Pam3CSK4 list also being identified in the LPS list (Figure 3C). Following LPS but not Pam3CSK4 stimulation, of note IL12B, IFNB1, IFNG, IL23A and CXCL10 were also significantly expressed as early as 1 hour (Figure 3C). There were a number of significantly expressed transcriptional regulators by 1 hour, with the majority of significantly expressed transcriptional regulators identified in the Pam3CSK4 1 hour list shared with LPS, including those of the NFκB family (NFKB1, NFKB2, NFKBIA, NFKBIE, NFKBIZ and REL) and the AP-1/CREB family (JUN, JUNB, ATF3, BATF3). Transcriptional regulators involved in cell development, proliferation and differentiation (BTG2, EGR3, ETV3, HHEX, MAFF, SKIL) and the post-transcriptional regulator ZFP36 (also known as Tristetraprolin) were also identified (Figure 3C).

### Upstream Analysis Identifies Potential Transcriptional Regulators

**Figure 4:**
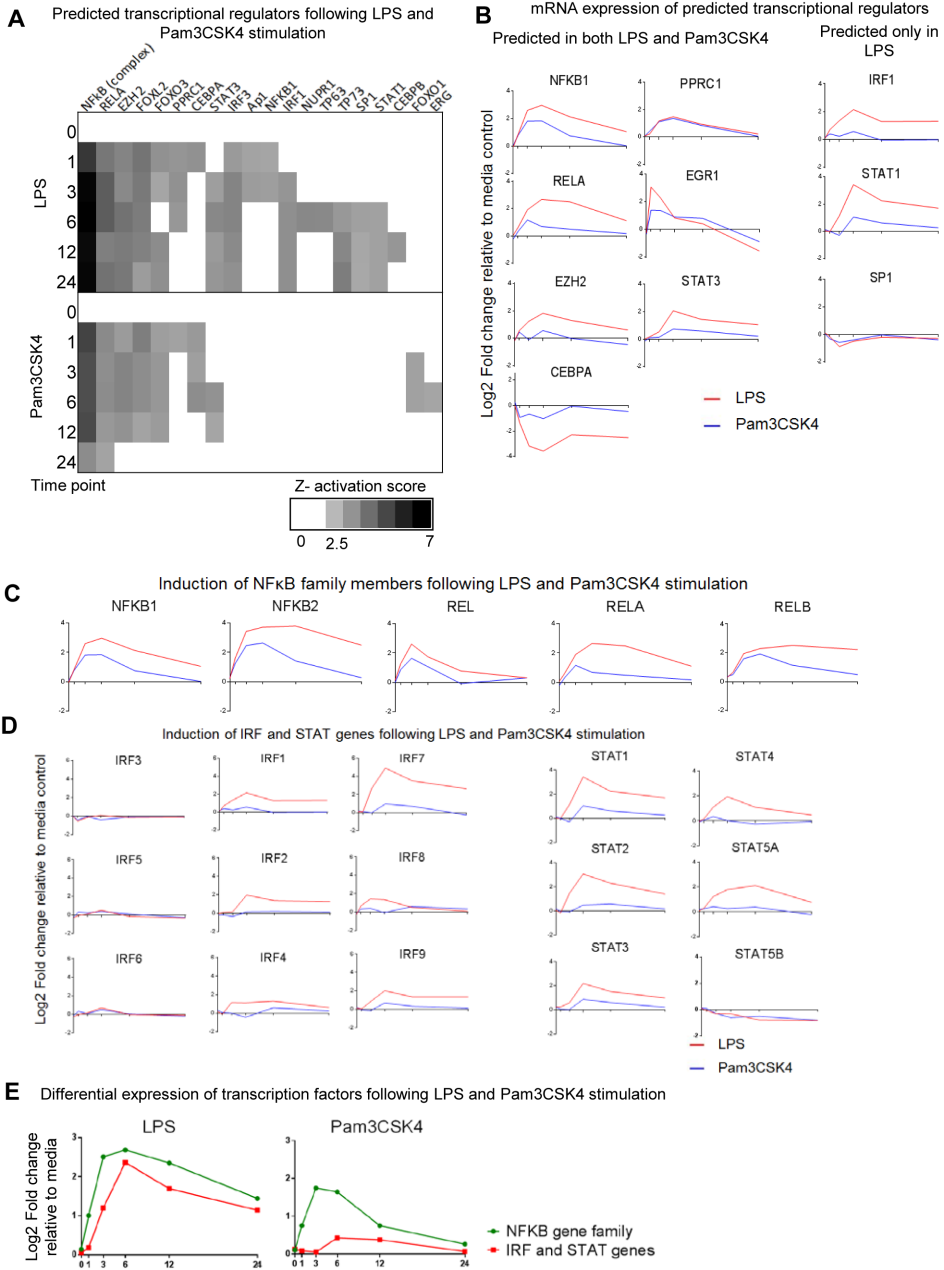
Predicted transcriptional regulator identification and NFκB, IRF and STAT gene expression following LPS and Pam3CSK4 stimulation. (**A**) Predicted upstream transcriptional regulators from IPA; stimulations analysed independently using Pam3CSK4 1202 and LPS 4777 transcripts lists, at each time point only genes whose expression were 1.8 FC different from the media control at that time point were taken into consideration. Predicted upstream transcription regulators which met the criteria (*p*<0.01 (Fishers Exact Test) and *z* activation score >2.5) shown plotted by *z*-activation score only at the time points where significance criteria met. (**B**) Mean mRNA expression of predicted transcription regulators plotted as log2 fold change (y axis) across time (x axis; 0, 1, 3, 6, 12 and 24 hours), fold change is relative to the media control at each time point, only those predicted transcription regulators whose mRNA expression is >1.8 FC relative to media control at one or more one time points are shown. (**C**) **NFkB genes.** Mean mRNA expression of the NFkB family genes, All 5 genes were significantly expressed and present in both Pam3CSK4 1202 and LPS 4777 transcript lists. (**D**) **Interferon Regulatory Factors.** Mean mRNA expression of the IRF and STAT genes. IRF1, IRF4, IRF7, IRF8, IRF9, STAT1, STAT2, STAT3, STAT4 and STAT5A were present in the LPS 4777 significantly expressed transcript list, none of the IRF or STAT genes (except STAT5B) were present in the 1202 Pam3CSK4 significant transcript list. (**E**) **Temporal Kinetics of NFkB and induced IRF and STAT genes.** Plotted for both LPS and Pam3CSK4 are mean fold change relative to media controls of the NFkB genes (NFKB1, NFKB2, REL, RELA, RELB) and mean fold change relative to media controls of selected IRF genes (IRF1, IRF2, IRF4, IRF7, IRF8, IRF9, STAT1, STAT2, STAT3, STAT4 and STAT5A).

In order to identify which transcriptional regulators may be responsible for the differences observed in gene expression between LPS and Pam3CSK4 stimulation we undertook upstream analysis of the gene lists at each time point within IPA. Upstream analysis attempts to predict which transcriptional regulator may be responsible for the observed differential gene expression by comparing the genes known to be regulated by a transcriptional regulator (derived from the literature) to those significantly differentially expressed genes identified at each time point from this analysis. It can be seen that the top predicted transcription regulator for both stimulations was the NFκB complex and that it was predicted to be activated from 1 hour onwards (Figure 4A).

By comparison of the mean mRNA expression of the predicted transcriptional regulators compared to media control it can be seen that the magnitude and temporal response was similar for LPS and Pam3CSK4 stimulation for PPRC1 and SP1 (Figure 4B). The predicted transcriptional regulators NFκB1, CEBPA, EGR1 shared similar mRNA temporal profiles between stimulations; however the magnitude of the response was greater upon LPS stimulation. RELA, EZH2 and STAT3 were predicted to be activated in both stimulations however had different temporal profiles between stimulations. RELA, EZH2 and STAT3 were upregulated earlier and to a greater magnitude in LPS compared to Pam3CSK4 and this upregulation persisted over time in the LPS stimulation compared to Pam3CSK4 stimulation. The mRNA expression of STAT1 and IRF1, which were only predicted to be activated following LPS stimulation, had different profiles being induced earlier and to a greater magnitude in LPS compared to Pam3CSK4 stimulations.

The NFκB genes REL, RELA, RELB, NFκB1 and NFκB2 were all significantly expressed and present in both LPS 4777 transcript and Pam3CSK4 1202 transcript lists (Figure 4C). Transcriptional regulators involved in IFN regulation IRF1, IRF3 and STAT1 were predicted to be activated in the LPS stimulation but not the Pam3CSK4 stimulation (Figure 4A). IRF1, IRF2, IRF4, IRF7, IRF8, IRF9, STAT1, STAT2, STAT3, STAT4 and STAT5A were all identified in the 4777 LPS and not the Pam3CSK4 1202 transcript list as being significantly differentially expressed, IRF7 having the greatest FC induction compared to media control. IRF3, IRF5 and IRF6 expression was not significantly regulated following LPS or Pam3CSK4 stimulation compared to media control (Figure 4D). Overall the inducible IRF and STAT gene expression level peaked later (6 hours) in expression at 6 hours compared to that of the NFκB family of genes which peaked between 3 to 6 hours (Figure 4E). We validated this difference between LPS and Pam3CSK4 in terms of the magnitude of expression at the 6 hour time point for NFKB1, NFKB2, STAT1, STAT2 and IRF7 by quantitative real time PCR (Figure S5). In addition the FC relative to the media control was lower for STAT1, STAT2 and IRF7 compared to the NFKB1 and NFKB2 following Pam3CSK4 stimulation.

### Upstream Analysis Identifies Potentially Active Cytokines

**Figure 5:**
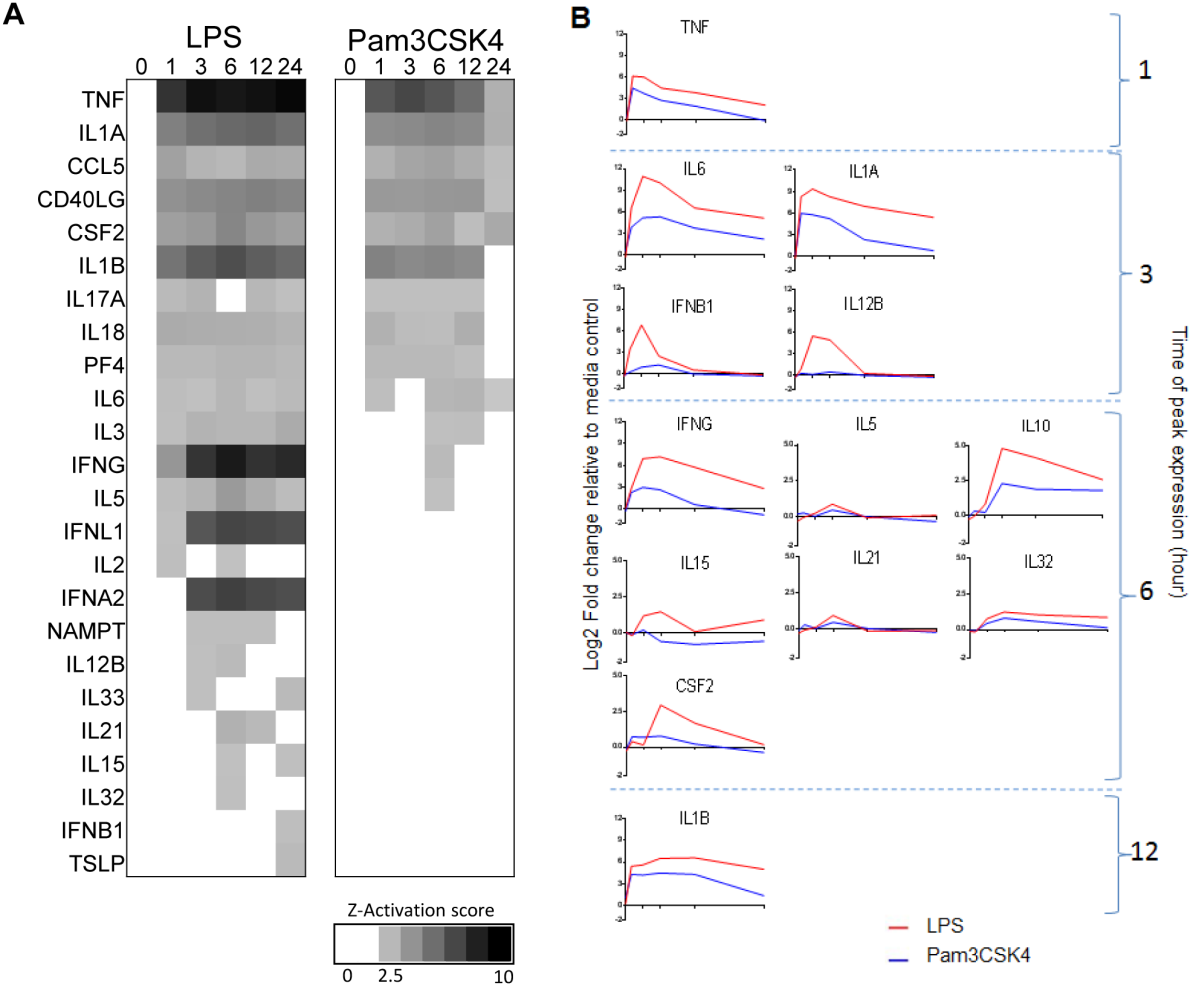
Identifying potential cytokines involved in autocrine gene regulation by upstream analysis within IPA. (**A**) Predicted activated cytokines from IPA upstream analysis; stimulations analysed independently using Pam3CSK4 1202 and LPS 4777 transcripts lists, at each time point only genes whose expression were 1.8 fold different from the media control at that time point were taken into consideration. Predicted upstream cytokines which met the criteria (*p* value <0.01 (Fishers Exact Test) and *z*- activation score >2.5) shown plotted by *z*-activation score only at the time points where significance criteria met. (**B**) Cytokine identified from either predicted upstream analysis or canonical pathway analysis (Fig. 3) mean mRNA expression plotted as log2 fold change (y axis) across time (x axis; 0, 1, 3, 6, 12 and 24 hours), fold change is relative to the media control at each time point, only those predicted cytokines whose mRNA expression is >1.8 fold upregulated relative to media control at one or more one time points are shown.

In order to attempt to identify the cytokines that were potentially involved in autocrine/paracrine signalling and subsequent gene expression regulation in response to LPS or Pam3CSK4 stimulation, we again undertook per-time point upstream analysis within IPA. TNF, IL1B and IL1A were predicted as potentially activated cytokines, and this predicted activation was early (by 1 hour) and sustained (Figure 5A). The mRNA expression of these cytokines compared to media control revealed them to be highly upregulated by 1 hour in both LPS and Pam3CSK4 stimulations. Although the magnitude was higher in LPS stimulation, they shared similar kinetic profile between stimulations (Figure 5B).

IL12B was predicted to be activated upon LPS stimulation but not Pam3CSK4 and this difference was observed in the mean mRNA expression of the IL12B gene (encoding for IL12p40) which was upregulated in response to LPS compared to media control and not following Pam3CSK4 stimulation (Figure 5). IFN cytokine activation was also only predicted in the LPS stimulation (IFNA2, IFNL1 and IFNB1), in keeping with this the kinetic profile of IFNB1 can be seen to be different between LPS and Pam3CSK4 stimulations. This difference between LPS and Pam3CSK4 stimulation was validated by real time PCR at the peak of IFNB1 expression at 3 hours. IL1A and IL6 which also peaked at 3 hours were seen to have a robust response following both LPS and Pam3CSK4 stimulation, although with a much greater response following LPS (Figure S5). IFNA and IFNL genes were of low magnitude expression (<1.8 FC different to media control) under both conditions of stimulations (not shown).

### IFN Gene Expression is Dominant Following LPS but not Pam3CSK4 Stimulation

Our data show that as early as 1 hour post stimulation IFN gene expression is seen to be upregulated following LPS but not Pam3CSK4 stimulation. This difference in IFN signalling was further emphasised by the *k*-means clustering, canonical pathway analysis and upstream analysis of both potential transcriptional regulators and cytokines highlighting a difference in IFN signalling following LPS and Pam3CSK4 stimulation.

IRF and STAT genes had been identified by *k*-means clustering, predicted upstream analysis, and canonical pathway analysis as being differentially activated between LPS and Pam3CSK4 stimulations.

To test if this difference in expression of IRF and STAT genes resulted in differential expression of IFN regulated genes we used a list of human type 1 IFN regulated genes generated from the Interferome database v2.0. We compared the expression of these type 1 IFN regulated genes between LPS and Pam3CSK4 stimulations. From this it can be seen that LPS stimulation results in a greater number of differentially regulated type 1 IFN genes compared to Pam3CSK4. In addition the magnitude of this differential regulation was much higher in LPS stimulated whole blood compared to Pam3CSK4 stimulated whole blood and the peak of this transcriptional response following both LPS and Pam3CSK4 was at 6 hours. (Figure S4).

### Induction of NFκB Signalling Pathway is Similar Following LPS and Pam3CSK4 Stimulation in Contrast to the IFN Signalling Pathway

**Figure 6:**
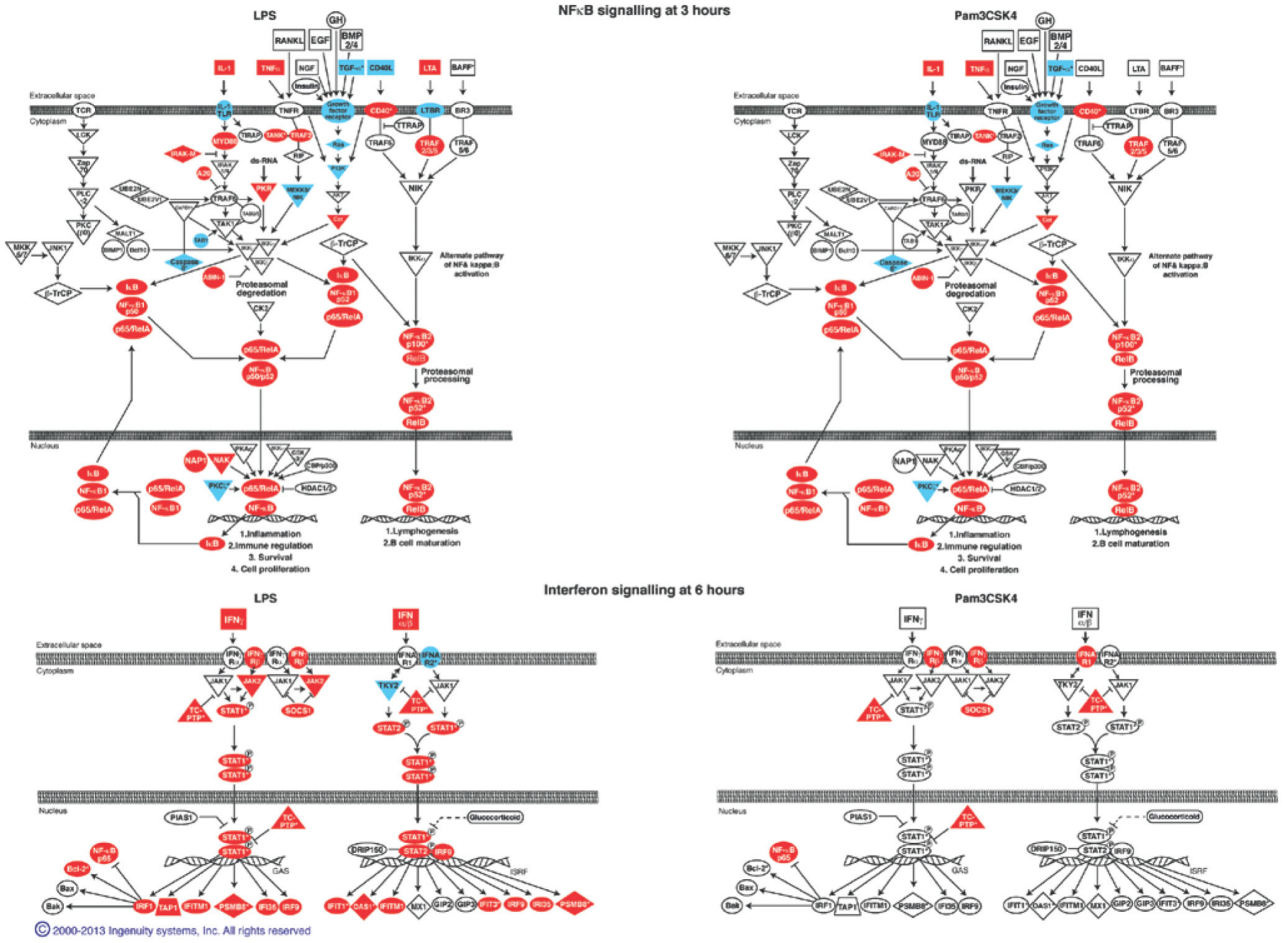
NFκB and Interferon signalling pathways. Shown at the peak of their significance, 3 and 6 hours respectively. Significantly expressed genes within the pathway (from Pam3CSK4 1202 and LPS 4777 lists) shaded red if upregulated or blue if down regulated.

The similarity in NFκB signalling and difference in IFN signalling following Pam3CSK4 and LPS stimulation is reflected in the IPA canonical pathways. There is similarity in the IPA canonical pathway “NFκB signalling” at 3 hours (the peak of significance for this pathway following LPS and Pam3CSK4 stimulations) following LPS and Pam3CSK4 stimulation. However there is clear difference IFN Signalling canonical pathway at 6 hours (the peak of significance for this pathway for LPS, the pathway is not significant at any time point following Pam3CSK4 stimulation) where there is seen to be difference following LPS and Pam3CSK4 stimulations (Figure 6).

### Comparison of Differential Transcriptional Expression Following Either *in* *vitro* Stimulation or *in* *vivo* LPS Stimulation of Human Whole Blood

**Figure 7:**
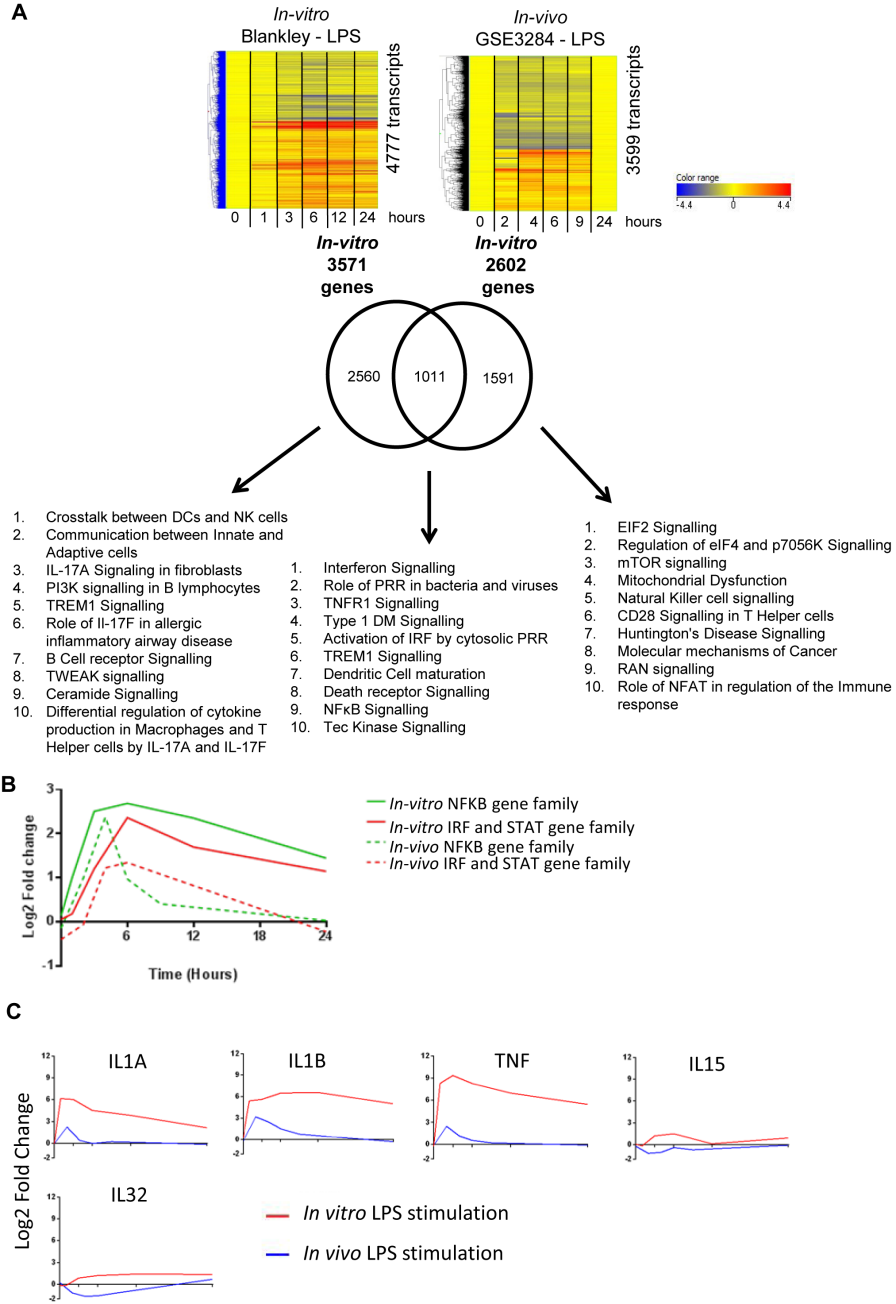
Comparison of Whole blood *in vitro* LPS stimulation and *in vivo* LPS stimulated blood leukocytes. GSE3284 data obtained from Gene Expression Omnibus (GEO) repository. Stimulations analysed independently to generate significant transcript lists: 4777 and 4791 significantly expressed transcripts respectively. Transcripts identified by normalising expression values to the median of the 0 hour samples, filtering by detection from background, statistical filtering (2 way ANOVA with Benjamini Hochberg multiple testing correction *p*<0.01) and retaining transcripts whose expression was greater than 1.8 FC different between the media control and stimulation samples at one or more time point. Heatmaps of significant transcript lists shown. Venn diagram of unique genes lists with canonical pathway analysis within IPA of subgroups (top ten significant pathways shown) (**B**) **Temporal Kinetics of NFkB and induced IRF and STAT genes.** For both *in vivo* and *in vitro* LPS stimulations mean fold change relative to controls of the NFkB genes (NFKB1, NFKB2, REL, RELA, RELB) and mean fold change relative to media controls of selected IRF genes (IRF1, IRF2, IRF4, IRF7, IRF8, IRF9, STAT1, STAT2, STAT3, STAT4 and STAT5A). (**C**) **Comparison of cytokine expression.** Mean mRNA expression of cytokines which were identified as being significant from the in vivo upstream analysis and were observed to be significantly expressed in both *in vivo* and *in vitro* datasets were plotted as log2 fold change (y axis) across time, fold change is relative to their controls at each time point.

*In vivo* LPS stimulated human whole blood data was obtained from the Gene Expression Omnibus (GSE3824 [16]), we analysed this data within Genespring (v12.6) using the same methodological principles used to analyse our own data. It can be seen that as opposed to *in vitro* LPS stimulation, following human *in vivo* LPS administration the transcriptional response had returned to baseline by 24 hours (Figure 7A). Comparative analysis of the significantly differentially regulated gene lists over 24 hours (per-time point analysis was not possible due to the different time points sampled, transcript level analysis not appropriate due to the difference in technology platforms), revealed that approximately 40% of the significantly differentially regulated genes following *in vivo* LPS administration were also differentially regulated following *in vitro* LPS stimulation. Canonical pathway analysis revealed these shared genes between stimulations were enriched for IFN, TNFR1, TREM and NFκB Signalling.

The genes differentially regulated following *in vivo* and not *in vitro* LPS stimulation were enriched for pathways involved in cell cycle, cellular development as well as immune function, however the most significant pathways for those genes differentially regulated following *in vitro* and not *in vivo* LPS stimulation were predominantly immune function pathways.

Transcriptional regulators which were differentially expressed following both *in vivo* and *in vitro* LPS stimulation included NFκB family genes (RELA, RELB, NFKB1, NKFB2) and IRF and STAT transcriptional regulators (IRF1, IRF2, IRF7, IRF8, IRF9, STAT1, STAT2, STAT3, STAT4, STAT5A) (Figure 7B). Analysis of the kinetic profile of these transcriptional regulators showed similar timing of the peak of induction for both *in vivo and in vitro*. Of note *in-vivo* expression then diminished soon after the peak of expression in contrast to that observed *in vitro* where persistent differential expression was observed still at 24 hours.

IL1A, IL1B, IL15, IL32 and TNF were cytokines which had been identified by *in vitro* upstream analysis and seen to be differentially expressed *in-vitro* and were also significantly differentially expressed following *in vivo* LPS administration (Figure 7C). However for IL1A, IL1B and TNF it can be seen that the magnitude of this induction is much lower *in vivo* than *in vitro* and that the kinetic profiles are different with expression returning to that of the media controls by 9 hours following stimulation *in vivo* in contrast to the *in vitro* experiment where expression remains elevated for the duration of the experiment compared to media controls.

The following cytokines/chemokines which had been predicted to be activated cytokines from the *in vitro* experiment and which were significantly differentially expressed *in vitro* but not *in vivo* were IFNB1, IFNG, CSF2, IL5, IL6, IL10, IL12B, and IL21.

## Discussion

A central goal of our study was to identify the key transcriptional differences between TLR4 and TLR2 ligation in a human whole blood system and demonstrate that in a whole blood system that the response to TLR stimulation can resemble that previously identified in isolated immune cells. We show that the magnitude of the transcriptional response both in terms of the number of differentially regulated genes as well as level of mRNA expression following LPS stimulation was greater and more sustained than the transcriptional response to Pam3CSK4. There was a common transcriptional response following stimulation with LPS and Pam3CSK4 which was dominated by NFκB regulated genes. In addition there was a separate IFN regulated transcriptional response (IRF and STAT mediated) seen mainly following LPS stimulation. This significant difference in the IFN response between TLR4 and TLR2 ligation could be seen as early as 1 hour post stimulation in our study, and reinforced in the later time points despite the potentially complex autocrine and paracrine signalling in the human whole blood system.

The common early transcriptional response following both LPS and Pam3CSK4 stimulations at 1 hour was characterised by highly expressed cytokines and chemokines (CCL2, CCL20, CCL23, CCL3, CCL3L1/CCCL3L3, CCL4, CXCL1, CXCL2, EBI3, EDN1, IL1A, IL1B, IL1RN, IL6, IL8 and TNF) as well as the significant upregulation of transcriptional regulators including the NFκB family (NFKB1, NFKB2, NFKBIA, NFKBIE, NFKBIZ and REL) and AP-1/CREB (JUN, JUNB, ATF3, BATF3). The gene ZFP36 which encodes for the protein Tristetraprolin was also identified as significantly up regulated by 1 hour which has been shown to act in a post-transcriptional regulatory role by binding to the mRNA of some cytokines and promoting their degradation [29]. The peak of expression compared to media control of NFκB genes was 3 to 6 hours following both LPS and Pam3CSK4 stimulation. By 24 hours following Pam3CSK4 stimulation the expression of these NFκB genes was not significantly different compared to media control, and similarly the expression of many proinflammatory cytokines (e.g. IL1A, IFNG and TNF) had diminished significantly by 24 hours compared to media control. This is in contrast to the expression following LPS stimulation where these NFκB genes and cytokines were still significantly upregulated compared to media control at 24 hours. This prolongation of the NFκB signalling seen following LPS stimulation maybe as a result of secondary induction of NFκB via the TRIF-TRAM adaptor molecules, or as a consequence of the IFN signalling [7], [8]. We also identified a common group of metallothionein genes (MT1G, MT1H, MT1E, MT1X, MT1M, MT1F) as being amongst the most up regulated genes at 24 hours following both LPS and Pam3CSK4 stimulation. Metallothionein genes are known to have a key role in oxidative stress and heavy metal detoxification [30]. It has also been shown that they may be induced by cytokines such as IL-6 and they are thought to have a gene regulatory role in inflammation [31]–[33]. The late induction of these genes in our system suggests that they may be being induced by subsequent auto/paracrine signalling downstream of the MyD88 adaptor.

Following LPS stimulation a differential transcriptional response of IFN regulated/regulatory genes was observed as compared to Pam3CSK4. This difference was detectable as early as 1 hour post LPS stimulation with the significant upregulation of the cytokine genes IFNB1 and IFNG and the transcriptional regulator IRF8 following LPS and not Pam3CSK4 stimulation. The IRF and STAT (IRF1, IRF2, IRF4, IRF7, IRF8, IRF9, STAT1, STAT2, STAT3, STAT4 and STAT5A) genes were seen to be differentially regulated following LPS stimulation and the peak of their expression was at 6 hours. This difference in induction of IRF and STAT transcription regulators following TLR4 and not TLR2 ligation is consistent with previous studies which have compared the temporal transcriptional response following TLR2 and TLR4 ligation in murine DCs and macrophages [10], [11]. The IFN signalling induced following TLR4 ligation and not TLR2 ligation can be accounted for by signalling through the MyD88 independent TRIF-TRAM, IRF3 pathway following TLR4 ligation resulting in induction of IFNβ, which then leads to positive feedback of IFN gene induction through induction of IRF7 amongst other IRFs [8]. IRF3 is thought to be constitutively expressed and its expression is not thought to be induced by TLR ligation, or type I or II IFN. This may explain why IRF3 was predicted by upstream analysis to be activated and its mRNA expression not significantly induced following TLR4 or TLR2 ligation in our study [8]. IRF7, which is known to be induced by type I IFN subsequent to IRF3 activation by TLR4 ligation was seen to be the most induced of the IRFs following TLR4 ligation [8]. This stepwise induction of the IRFs may account for the later peak of expression at 6 hours of the inducible IRF and STAT genes compared to the NFκB genes which peaked at 3 hours in our study. This later peak in transcription factors associated with IFN regulation compared to NFκB genes is in agreement with studies in murine macrophages as well as *ex-vivo* human blood leukocytes. Slight differences in the timing of peaks observed in our study and these other studies could potentially be explained by the difference in time points sampled or differences in the systems used [10], [34].

Following LPS stimulation the peak of differential transcription compared to media control was at 6 hours in terms of the number of differentially regulated genes. At 24 hours 1443 genes were still significantly differentially regulated. This persistence of differential transcription at 24 hours is observed in human LPS stimulated *in vitro* transcriptional studies [12], [13]. However this in contrast to that noted in human *in-vivo* stimulation where transcriptional difference peaked between 4 to 6 hours and by 24 hours the transcriptional signature had returned to baseline [16]. Several cytokines/chemokines were not seen to be significantly induced *in vivo* compared to *in *vitro and these differences in expression and persistence of differential expression observed *in vitro compared to in vivo* could be explained by trafficking/removal of activated immune cells out of the circulation *in vivo*, which is not possible to be represented in an *in vitro* whole blood system.

The ability to utilise *in vitro* stimulated human whole blood for transcriptomic analysis of the early innate immune response has potential advantages over the use of isolated immune cells as whole blood stimulation can be carried out in a laboratory where the expertise or equipment to isolate immune cells from blood is lacking. In addition the volume of blood needed for stimulation is much less than that required to isolate immune cells, meaning experiments can be performed in populations where access to larger volumes of blood is not possible e.g. paediatrics or it could be possible to undertake more stimulations/time points with a given volume. The results however must be interpreted in the context of the complex autocrine/paracrine interactions which occur in a mixed cell culture and therefore stimulation of isolated immune cells will remain advantageous to interrogate in detail a specific transcriptional response.

We have shown that human whole blood can be used to study the early temporal transcriptional response following TLR2 and TLR4 ligation and that the results obtained are comparable to those from isolated murine and human immune cells.

## Supporting Information

Figure S1 **Activation of samples by culture conditions. (A)** Heatmap of expression, clustered by transcripts, shows that 2 individuals (out of 6) media controls show activation (marked with arrows), the genes differentially expressed in these 2 media control samples are similar to LPS, but lower in magnitude. Transcripts identified by normalisation to 0 hour samples, filtering by detection from background, statistical filtering (2 way ANOVA with Benjamini Hochberg p<0.01) and then transcripts retained whose expression was >1.8 FC from another condition. **(B)** Activation is not due to length of time in transport conditions, nor is it individual specific. The single individual shown here had not previously activated, and was included in final dataset. Blood was collected at time point 0 and left in sealed vacutainers, the vacutainers were opened at one hour intervals and 1 ml human whole blood was either immediately mixed with Tempus solution (labelled as Direct from vacutainer) or plated for 3 hours with either media control (RPMI-1640 with GlutaMAX) or LPS (1 ng/ml) and then mixed with Tempus solution. Reagents and containers (including vacutainers) are endotoxin free (undetectable by Limulus assay - sensitivity <0.03 EU/ml). Heatmap of expression (duplicate stimulations from the same individual shown, 2619 transcripts), clustered by transcripts shows that regardless of length of time in vacutainer activation occurred in all media control samples, and is not observed in the direct from vacutainer samples, implying that activation is dependent on culture conditions and is not a function of length of time *ex-vivo* or length of time spent in the vacutainers. Transcripts identified by normalisation to median of “Direct from vacutainer” samples, filtering by detection from background, statistical filtering (ANOVA with Benjamini Hochberg *p*<0.01) and then transcripts retained whose expression was >1.8 FC from another condition.
(TIF)

Figure S2 **Transcriptional changes in media controls over time.** **(A)** Heatmap of normalised expression values of 377 transcripts which were identified to be significantly differentially expressed overtime (transcripts identified by normalisation to 0 hour samples, filtering by detection from background, statistical filtering (One way ANOVA with Benjamini Hochberg p<0.01) and then transcripts retained whose expression was >1.8 FC from the 0 hour samples. Plotted above the heatmaps is the number of significantly expressed genes (mapped in IPA) that were differentially expressed at each time point by more than 1.8 FC compared to the 0 hour samples. **(B)** Top ten IPA canonical pathways of the significantly expressed genes at 24 hours, with the –log *p* value for the pathway and the significantly differentially expressed genes listed for each pathway.
(TIF)

Figure S3 **Metallothionein gene expression. (A)** Heatmap of averaged Metallothionein mRNA expression over time following LPS or Pam3CSK4 stimulation, values normalised to the median of the 0 hour. Note asynchronous scale.
(TIF)

Figure S4 **Interferon regulated genes.** Heatmap of averaged expression values of Type 1 Interferon regulated genes (List obtained from Interferome v2.0), normalised to the median of the 0 hour, genes retained if they were expressed greater than 1.8 FC from media control in at least one stimulation in one or more time points (resulting in 1105 genes). Graphed above heatmap is the mean absolute fold change of these Type 1 interferon regulated genes.
(TIF)

Figure S5 **Real time PCR.** Real time PCR of selected genes following LPS and Pam3CSK4 stimulations and media controls, normalised to GAPDH expression. Mean fold change calculated between media controls and stimulations.
(TIF)

Table S1 **Volunteer whole blood composition measured by Celltac Automated Hematology Analyzer (MEK-6400J/K, Nihon Kohden) at time point 0 hour.**
(TIF)

Table S2 **Pearson correlations for k-means derived clusters from** **Figure 2** **.**
(TIF)

File S1 **Listings of transcripts, LPS k-means clusters from** **Figure 2** **.**
(XLSX)

File S2 **Listings of transcripts, Pam3CSK4 k-means clusters from** **Figure 2** **.**
(XLSX)

File S3 **LPS time course data.** Listings of transcripts from 4777 significant transcript list whose mean expression was 1.8 FC different to media control at each time point from Figure 3.
(XLSX)

File S4 **Pam3CSK4 time course data.** Listings of transcripts from 1202 significant transcript list whose mean expression was 1.8 FC different to media control at each time point from Figure 3.
(XLSX)

## References

[1] Pascual V, Chaussabel D, Banchereau J (2010) A genomic approach to human autoimmune diseases. Annu Rev Immunol 28: 535–571. 20192809 10.1146/annurev-immunol-030409-101221 PMC2847838

[2] Germain RN, Meier-Schellersheim M, Nita-Lazar A, Fraser ID (2011) Systems biology in immunology: a computational modeling perspective. Annu Rev Immunol 29: 527–585. 21219182 10.1146/annurev-immunol-030409-101317 PMC3164774

[3] Khatri P, Sirota M, Butte AJ (2012) Ten years of pathway analysis: current approaches and outstanding challenges. PLoS Comput Biol 8: e1002375. 22383865 10.1371/journal.pcbi.1002375 PMC3285573

[4] Zak DE, Aderem A (2009) Systems biology of innate immunity. Immunol Rev 227: 264–282. 19120490 10.1111/j.1600-065X.2008.00721.x PMC2697920

[5] Moresco EM, LaVine D, Beutler B (2011) Toll-like receptors. Curr Biol 21: R488–493. 21741580 10.1016/j.cub.2011.05.039

[6] Kawai T, Akira S (2010) The role of pattern-recognition receptors in innate immunity: update on Toll-like receptors. Nat Immunol 11: 373–384. 20404851 10.1038/ni.1863

[7] Medzhitov R, Horng T (2009) Transcriptional control of the inflammatory response. Nat Rev Immunol 9: 692–703. 19859064 10.1038/nri2634

[8] Honda K, Taniguchi T (2006) IRFs: master regulators of signalling by Toll-like receptors and cytosolic pattern-recognition receptors. Nat Rev Immunol 6: 644–658. 16932750 10.1038/nri1900

[9] Ramirez-Carrozzi VR, Nazarian AA, Li CC, Gore SL, Sridharan R, et al (2006) Selective and antagonistic functions of SWI/SNF and Mi-2beta nucleosome remodeling complexes during an inflammatory response. Genes Dev 20: 282–296. 16452502 10.1101/gad.1383206 PMC1361700

[10] Elkon R, Linhart C, Halperin Y, Shiloh Y, Shamir R (2007) Functional genomic delineation of TLR-induced transcriptional networks. BMC Genomics 8: 394. 17967192 10.1186/1471-2164-8-394 PMC2175519

[11] Amit I, Garber M, Chevrier N, Leite AP, Donner Y, et al (2009) Unbiased reconstruction of a mammalian transcriptional network mediating pathogen responses. Science 326: 257–263. 19729616 10.1126/science.1179050 PMC2879337

[12] Roach JC, Smith KD, Strobe KL, Nissen SM, Haudenschild CD, et al (2007) Transcription factor expression in lipopolysaccharide-activated peripheral-blood-derived mononuclear cells. Proc Natl Acad Sci U S A 104: 16245–16250. 17913878 10.1073/pnas.0707757104 PMC2042192

[13] Gilchrist M, Thorsson V, Li B, Rust AG, Korb M, et al (2006) Systems biology approaches identify ATF3 as a negative regulator of Toll-like receptor 4. Nature 441: 173–178. 16688168 10.1038/nature04768

[14] Litvak V, Ramsey SA, Rust AG, Zak DE, Kennedy KA, et al (2009) Function of C/EBPdelta in a regulatory circuit that discriminates between transient and persistent TLR4-induced signals. Nat Immunol 10: 437–443. 19270711 10.1038/ni.1721 PMC2780024

[15] Ramsey SA, Klemm SL, Zak DE, Kennedy KA, Thorsson V, et al (2008) Uncovering a macrophage transcriptional program by integrating evidence from motif scanning and expression dynamics. PLoS Comput Biol 4: e1000021. 18369420 10.1371/journal.pcbi.1000021 PMC2265556

[16] Calvano SE, Xiao W, Richards DR, Felciano RM, Baker HV, et al (2005) A network-based analysis of systemic inflammation in humans. Nature 437: 1032–1037. 16136080 10.1038/nature03985

[17] Chung TP, Laramie JM, Meyer DJ, Downey T, Tam LH, et al (2006) Molecular diagnostics in sepsis: from bedside to bench. J Am Coll Surg 203: 585–598. 17084318 10.1016/j.jamcollsurg.2006.06.028 PMC7118893

[18] de Kleijn S, Kox M, Sama IE, Pillay J, van Diepen A, et al (2012) Transcriptome kinetics of circulating neutrophils during human experimental endotoxemia. PLoS One 7: e38255. 22679495 10.1371/journal.pone.0038255 PMC3367952

[19] Nguyen TT, Foteinou PT, Calvano SE, Lowry SF, Androulakis IP (2011) Computational identification of transcriptional regulators in human endotoxemia. PLoS One 6: e18889. 21637747 10.1371/journal.pone.0018889 PMC3103499

[20] Aguillon JC, Escobar A, Ferreira V, Aguirre A, Ferreira L, et al (2001) Daily production of human tumor necrosis factor in lipopolysaccharide (LPS)-stimulated ex vivo blood culture assays. Eur Cytokine Netw 12: 105–110. 11282553

[21] Wilson BM, Severn A, Rapson NT, Chana J, Hopkins P (1991) A convenient human whole blood culture system for studying the regulation of tumour necrosis factor release by bacterial lipopolysaccharide. J Immunol Methods 139: 233–240. 1904465 10.1016/0022-1759(91)90193-j

[22] Mayringer I, Reindl M, Berger T (2000) A critical comparison of frequently used methods for the analysis of tumor necrosis factor-alpha expression by human immune cells. J Immunol Methods 235: 33–40. 10675755 10.1016/s0022-1759(99)00208-2

[23] De Groote D, Zangerle PF, Gevaert Y, Fassotte MF, Beguin Y, et al (1992) Direct stimulation of cytokines (IL-1 beta, TNF-alpha, IL-6, IL-2, IFN-gamma and GM-CSF) in whole blood. I. Comparison with isolated PBMC stimulation. Cytokine 4: 239–248. 1498259 10.1016/1043-4666(92)90062-v

[24] Netea MG, Drenth JP, De Bont N, Hijmans A, Keuter M, et al (1996) A semi-quantitative reverse transcriptase polymerase chain reaction method for measurement of MRNA for TNF-alpha and IL-1 beta in whole blood cultures: its application in typhoid fever and exentric exercise. Cytokine 8: 739–744. 8932986 10.1006/cyto.1996.0098

[25] Chen J, Bruns AH, Donnelly HK, Wunderink RG (2010) Comparative in vitro stimulation with lipopolysaccharide to study TNFalpha gene expression in fresh whole blood, fresh and frozen peripheral blood mononuclear cells. J Immunol Methods 357: 33–37. 20307542 10.1016/j.jim.2010.03.006

[26] Wurfel MM, Park WY, Radella F, Ruzinski J, Sandstrom A, et al (2005) Identification of high and low responders to lipopolysaccharide in normal subjects: an unbiased approach to identify modulators of innate immunity. J Immunol 175: 2570–2578. 16081831 10.4049/jimmunol.175.4.2570

[27] Obermoser G, Presnell S, Domico K, Xu H, Wang Y, et al (2013) Systems scale interactive exploration reveals quantitative and qualitative differences in response to influenza and pneumococcal vaccines. Immunity 38: 831–844. 23601689 10.1016/j.immuni.2012.12.008 PMC3681204

[28] Rusinova I, Forster S, Yu S, Kannan A, Masse M, et al (2013) Interferome v2.0: an updated database of annotated IFN-regulated genes. Nucleic Acids Res 41: D1040–1046. 23203888 10.1093/nar/gks1215 PMC3531205

[29] Anderson P, Phillips K, Stoecklin G, Kedersha N (2004) Post-transcriptional regulation of proinflammatory proteins. J Leukoc Biol 76: 42–47. 15075353 10.1189/jlb.1103536

[30] Ruttkay-Nedecky B, Nejdl L, Gumulec J, Zitka O, Masarik M, et al (2013) The role of metallothionein in oxidative stress. Int J Mol Sci 14: 6044–6066. 23502468 10.3390/ijms14036044 PMC3634463

[31] Wu C, Pot C, Apetoh L, Thalhamer T, Zhu B, et al (2013) Metallothioneins negatively regulate IL-27-induced type 1 regulatory T-cell differentiation. Proc Natl Acad Sci U S A 110: 7802–7807. 23630250 10.1073/pnas.1211776110 PMC3651423

[32] Abdel-Mageed AB, Agrawal KC (1998) Activation of nuclear factor kappaB: potential role in metallothionein-mediated mitogenic response. Cancer Res 58: 2335–2338. 9622069

[33] Lee DK, Carrasco J, Hidalgo J, Andrews GK (1999) Identification of a signal transducer and activator of transcription (STAT) binding site in the mouse metallothionein-I promoter involved in interleukin-6-induced gene expression. Biochem J 337 (Pt 1): 59–65. PMC1219936 9854025

[34] Seok J, Xiao W, Moldawer LL, Davis RW, Covert MW (2009) A dynamic network of transcription in LPS-treated human subjects. BMC Syst Biol 3: 78. 19638230 10.1186/1752-0509-3-78 PMC2729748

