# Supplementary figures and images for: Identification of the Key Differential Transcriptional Responses of Human Whole Blood Following TLR2 or TLR4 Ligation In-Vitro
Source: PLoS One. 2014 May 19;9(5):e97702. doi: 10.1371/journal.pone.0097702 (PMC4026482; doi:10.1371/journal.pone.0097702)

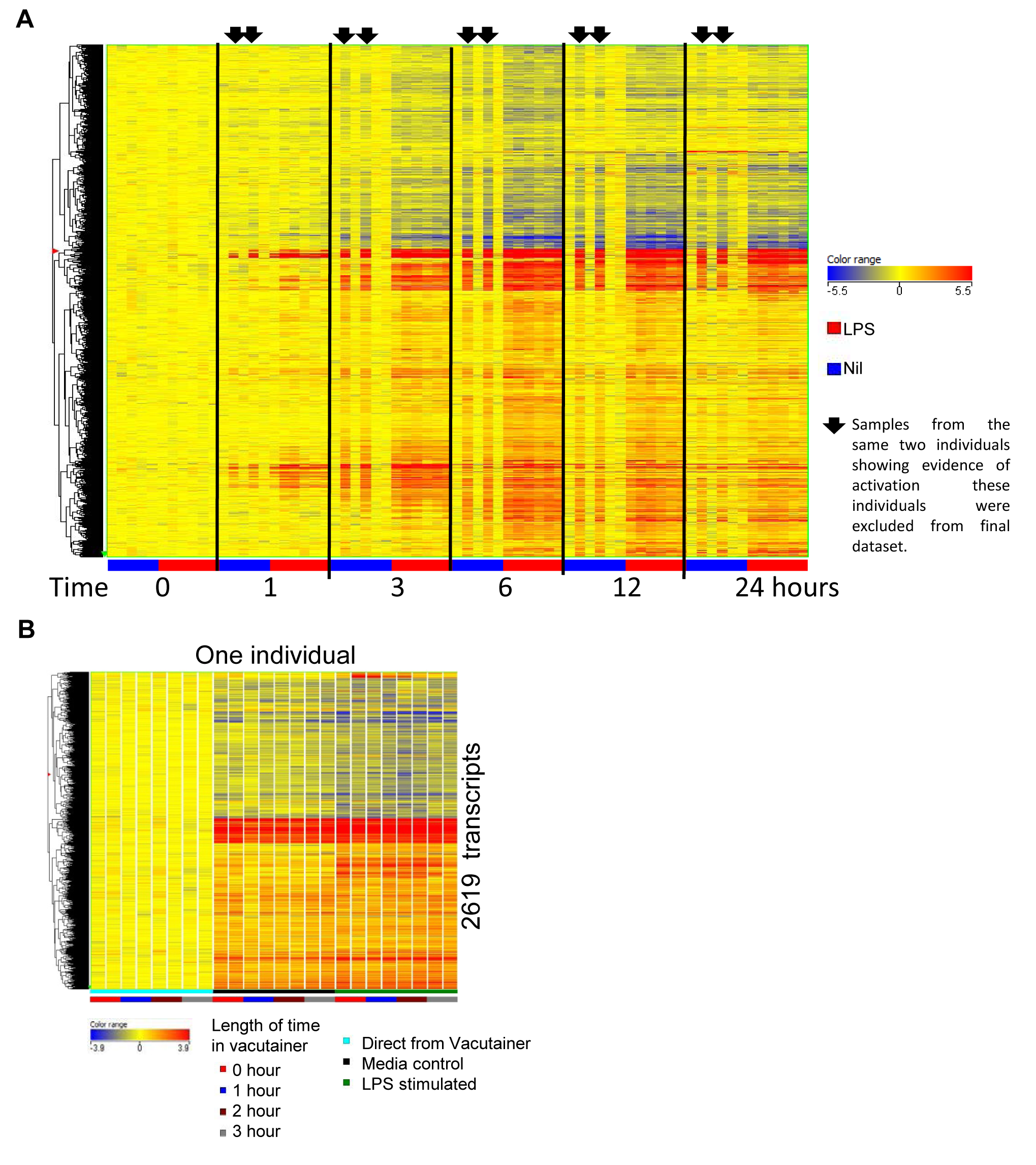

Supplement: Figure S1 — Activation of samples by culture conditions. (A) Heatmap of expression, clustered by transcripts, shows that 2 individuals (out of 6) media controls show activation (marked with arrows), the genes differentially expressed in these 2 media control samples are similar to LPS, but lower in magnitude. Transcripts identified by normalisation to 0 hour samples, filtering by detection from background, statistical filtering (2 way ANOVA with Benjamini Hochberg p<0.01) and then transcripts retained whose expression was >1.8 FC from another condition. (B) Activation is not due to length of time in transport conditions, nor is it individual specific. The single individual shown here had not previously activated, and was included in final dataset. Blood was collected at time point 0 and left in sealed vacutainers, the vacutainers were opened at one hour intervals and 1 ml human whole blood was either immediately mixed with Tempus solution (labelled as Direct from vacutainer) or plated for 3 hours with either media control (RPMI-1640 with GlutaMAX) or LPS (1 ng/ml) and then mixed with Tempus solution. Reagents and containers (including vacutainers) are endotoxin free (undetectable by Limulus assay - sensitivity <0.03 EU/ml). Heatmap of expression (duplicate stimulations from the same individual shown, 2619 transcripts), clustered by transcripts shows that regardless of length of time in vacutainer activation occurred in all media control samples, and is not observed in the direct from vacutainer samples, implying that activation is dependent on culture conditions and is not a function of length of time ex-vivo or length of time spent in the vacutainers. Transcripts identified by normalisation to median of “Direct from vacutainer” samples, filtering by detection from background, statistical filtering (ANOVA with Benjamini Hochberg p<0.01) and then transcripts retained whose expression was >1.8 FC from another condition. (TIF) [file pone.0097702.s001.tif]

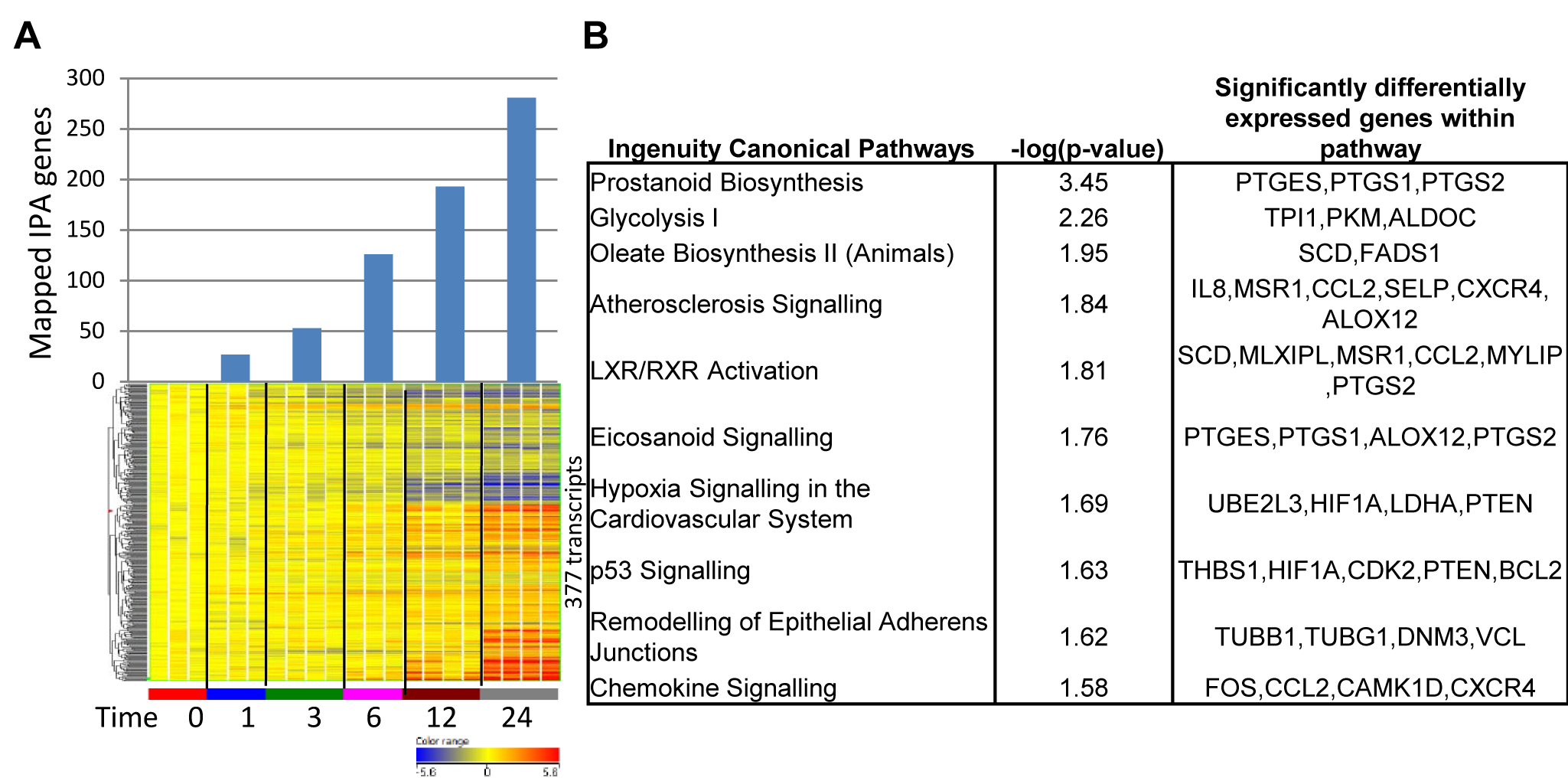

Supplement: Figure S2 — Transcriptional changes in media controls over time. (A) Heatmap of normalised expression values of 377 transcripts which were identified to be significantly differentially expressed overtime (transcripts identified by normalisation to 0 hour samples, filtering by detection from background, statistical filtering (One way ANOVA with Benjamini Hochberg p<0.01) and then transcripts retained whose expression was >1.8 FC from the 0 hour samples. Plotted above the heatmaps is the number of significantly expressed genes (mapped in IPA) that were differentially expressed at each time point by more than 1.8 FC compared to the 0 hour samples. (B) Top ten IPA canonical pathways of the significantly expressed genes at 24 hours, with the –log p value for the pathway and the significantly differentially expressed genes listed for each pathway. (TIF) [file pone.0097702.s002.tif]

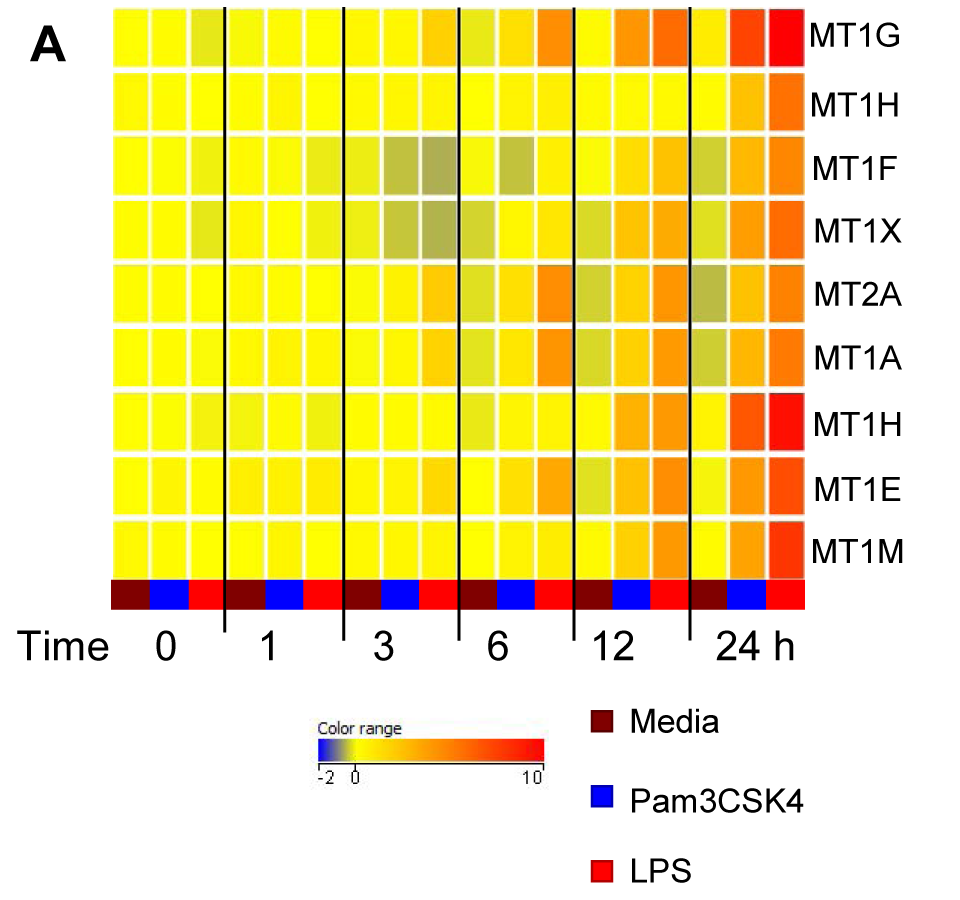

Supplement: Figure S3 — Metallothionein gene expression. (A) Heatmap of averaged Metallothionein mRNA expression over time following LPS or Pam3CSK4 stimulation, values normalised to the median of the 0 hour. Note asynchronous scale. (TIF) [file pone.0097702.s003.tif]

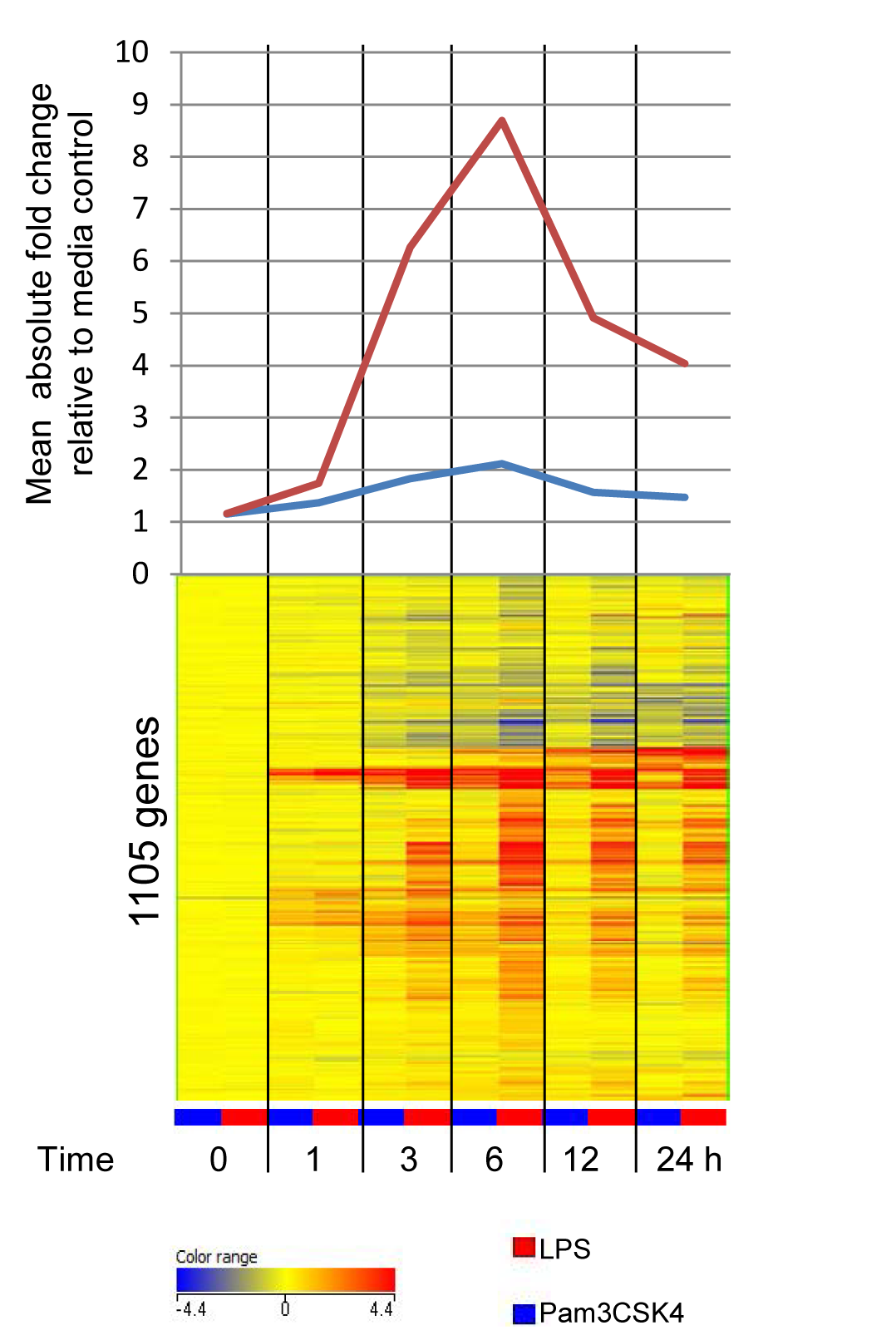

Supplement: Figure S4 — Interferon regulated genes. Heatmap of averaged expression values of Type 1 Interferon regulated genes (List obtained from Interferome v2.0), normalised to the median of the 0 hour, genes retained if they were expressed greater than 1.8 FC from media control in at least one stimulation in one or more time points (resulting in 1105 genes). Graphed above heatmap is the mean absolute fold change of these Type 1 interferon regulated genes. (TIF) [file pone.0097702.s004.tif]

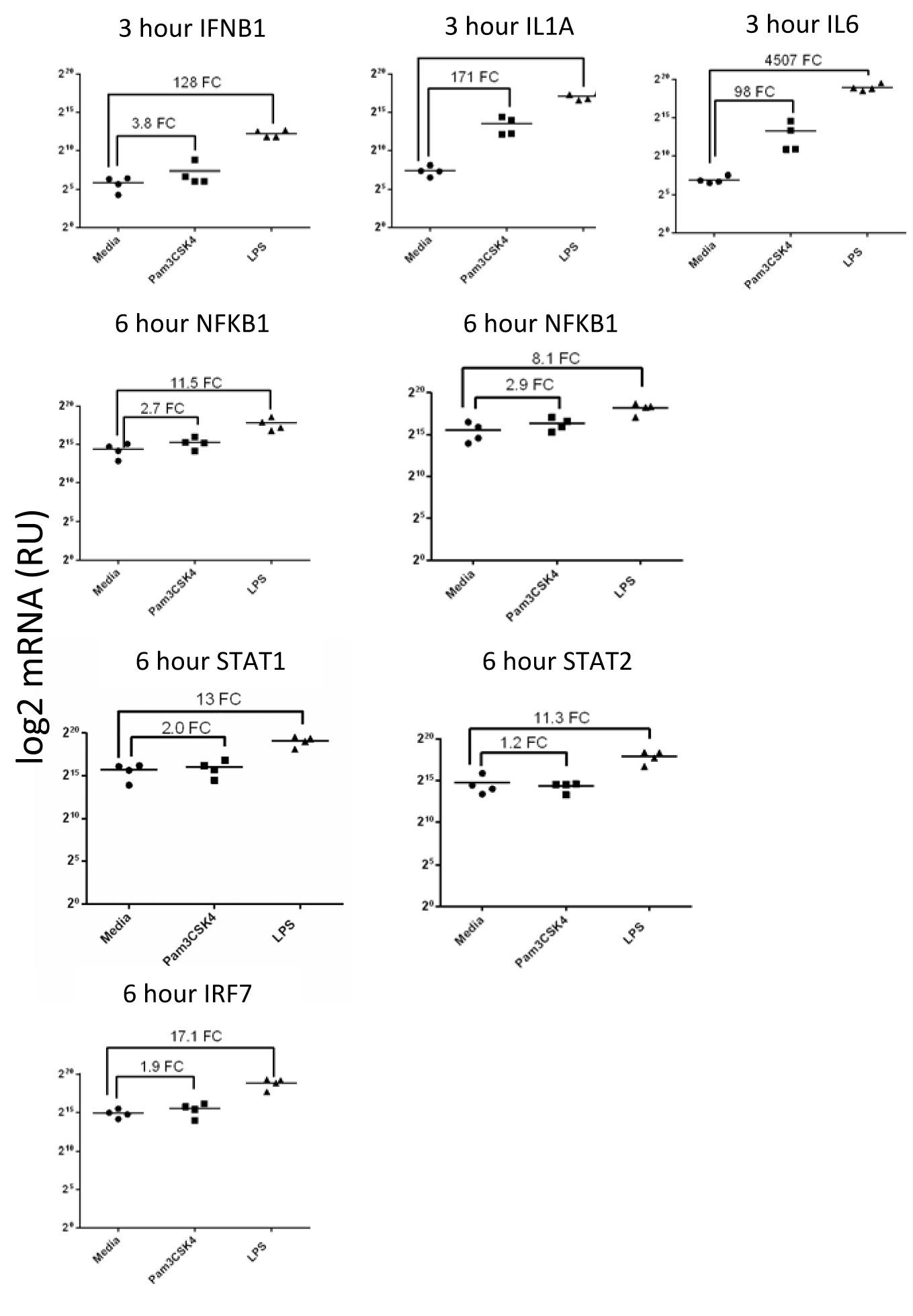

Supplement: Figure S5 — Real time PCR. Real time PCR of selected genes following LPS and Pam3CSK4 stimulations and media controls, normalised to GAPDH expression. Mean fold change calculated between media controls and stimulations. (TIF) [file pone.0097702.s005.tif]

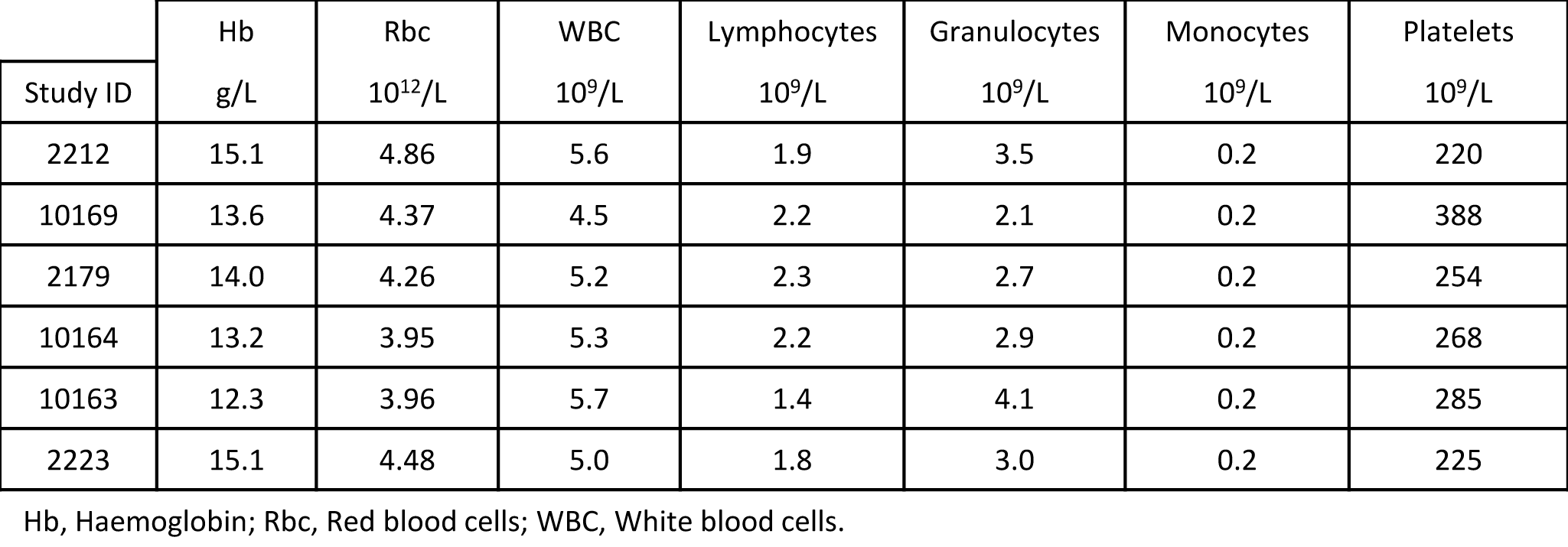

Supplement: Table S1 — Volunteer whole blood composition measured by Celltac Automated Hematology Analyzer (MEK-6400J/K, Nihon Kohden) at time point 0 hour. (TIF) [file pone.0097702.s006.tif]

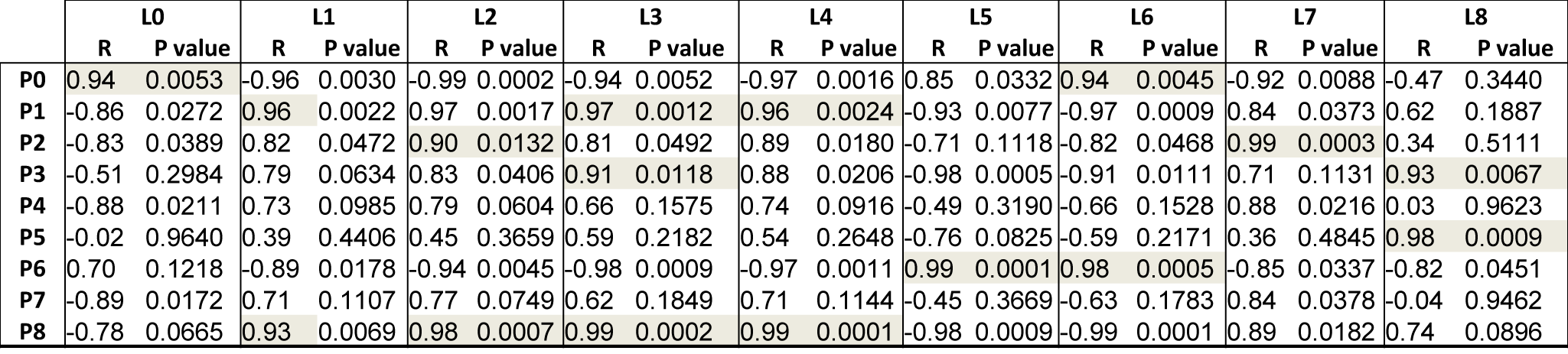

Supplement: Table S2 — Pearson correlations for k-means derived clusters from Figure 2 . (TIF) [file pone.0097702.s007.tif]
